# Spatiotemporal characteristics of P-selectin-induced β_2_ integrin activation of human neutrophils under flow

**DOI:** 10.3389/fimmu.2022.1023865

**Published:** 2022-11-10

**Authors:** Xiaoxi Sun, Bing Huang, Yuping Pan, Jinhua Fang, Hefeng Wang, Yanru Ji, Yingchen Ling, Pei Guo, Jiangguo Lin, Quhuan Li, Ying Fang, Jianhua Wu

**Affiliations:** ^1^ School of Biology and Biological Engineering, South China University of Technology, Guangzhou, China; ^2^ Guangdong Provincial Key Laboratory of Gastroenterology, Institute of Gastroenterology of Guangdong Province, Department of Gastroenterology, Nanfang Hospital, Southern Medical University, Guangzhou, China; ^3^ Research Center of Medical Sciences, Guangdong Provincial People’s Hospital, Guangdong Academy of Medical Sciences, Guangzhou, China

**Keywords:** neutrophils, P-selectin, LFA-1, Mac-1, signaling pathway, shear stress

## Abstract

Activation of integrins is crucial for recruitment of flowing leukocytes to inflammatory or injured vascular sites, but their spatiotemporal characteristics are incompletely understood. We discovered that β_2_-integrin activation over the entire surface of neutrophils on immobilized P-selectin occurred *via* mitogen-activated protein kinase (MAPK) or non-MAPK signaling with a minute-level timescale in a force-dependent manner. In flow, MAPK signaling required intracellular Ca^2+^ release to activate integrin within 2 min. Integrin activation *via* non-MAPK signaling occurred first locally in the vicinity of ligated P-selectin glycoprotein ligand-1 (PSGL-1) within sub-seconds, and then over the entire cell surface within 1 min in an extracellular Ca^2+^ influx-dependent manner. The transition from a local (but rapid) to global (but slow) activation mode was triggered by ligating the freshly activated integrin. Lipid rafts, moesin, actin, and talin were involved in non-MAPK signaling. Fluid loads had a slight effect on local integrin activation with a second-level timescale, but served as enhancers of global integrin activation.

## Introduction

Leukocytes are key actors in a series of physiological and pathological processes: hemostasis, thrombosis, and cancer metastasis (1, 2). Recruitment of “flowing” leukocytes to inflammatory or injury sites on vascular surfaces involves a multistep cascade consisting of tethering, rolling, firm adhesion, crawling, and transmigration of cells (1, 3). The dominant mediators for these events are selectins. They mediate the tethering to, and rolling of, cells on the activated endothelium or platelet surfaces. β_2_ integrins are responsible for slow rolling and firm adhesion (1, 4, 5). Activation of β_2_ integrins is a key (but early) event in leukocyte recruitment (6, 7). Selectin-mediated cell rolling initiates activation of β_2_ integrins, which then regulate subsequent slow rolling and firm adhesion (8–10). The spatiotemporal characteristics of activation of β_2_ integrins should be assignable in β_2_ integrin-involved cell–cell and cell–extracellular matrix interactions (11).

L-selectin is expressed on leukocytes. P- and E-selectin are expressed on activated platelets and endothelial cells. These three members of the selectin family are receptors of P-selectin glycoprotein ligand-1 (PSGL-1) (12, 13). Lymphocyte function-associated antigen-1 (LFA-1; also called α_L_β_2_ or cluster of differentiation (CD11a/CD18) and macrophage antigen-1 (Mac-1; also known as α_M_β_2_ and CD11b/CD18) are β_2_ integrins whose ligands are intercellular adhesion molecule-1 (ICAM-1) on activated endothelial cells and platelet glycoprotein (GP) Ibα (5, 11, 14). LFA-1 mediates the slow rolling and firm adhesion of flowing neutrophils, but Mac-1 governs the subsequent intraluminal crawling (3, 12).

Like other integrins, β_2_ integrins have “low”, “moderate”, and “high” affinity states corresponding to three distinct conformations: bent, extended, and head-opened (15). Activation of β_2_ integrins involves a transition from a low to higher affinity state, and arises from a transformation from a bent or head-closed conformation to an extended or head-open conformation (6). Recent work suggests a new bent but head-open conformation of β_2_ integrins that limits neutrophil adhesion through binding with ICAM-1 in *cis* isomerism instead of *trans* isomerism (7).

β_2_ integrins can be activated by Mg^2+^, Mn^2+^, chemokines, and adhesion molecules, usually in a force-dependent manner (16–18). In flow, P- and E-selectins mediate cell rolling by binding with PSGL-1 and then induce activation of leukocyte β_2_ integrins and, almost synchronously, activated β_2_ integrins slow-down cell rolling by binding with ICAM-1 (10). ICAM-1 engagement reduces neutrophil rolling on the P-selectin-coated substrate significantly to support neutrophil arrest, especially in the presence of interleukin-8 (IL-8) (19). Before and after cell arrest, small-sized clusters of β_2_ integrins in a high-affinity state form on the neutrophil surface, unlike the nearly homogeneous distribution of LFA-1 on the cell surface (7). The activated LFA-1 clusters on the interface between neutrophils and endothelial cells take a broad patch-like form with indistinct outlines and adhere within a few minutes (20). This observation suggests that activation of β_2_-integrin signaling in leukocytes is instantaneous for cell rolling and early adhesion, but not in the late stage of cell adhesion. This instantaneous activation of signaling is also required for leukocyte arrest mediated by endothelium-bound chemokines (7, 18).

Selectin-mediated LFA-1 activation in neutrophils is believed to be *via* the mitogen-activated protein kinase (MAPK) signaling pathway. The latter comprises an Src family tyrosine kinase (Hck and Lyn, or Fgr), an immunoreceptor tyrosine-based activation motif (ITAM)-containing protein [Fc receptor γ-chain (FcRγ) or DNAX-activating protein of 12kDa (DAP12)] and tyrosine kinase (Syk) in the spleen (21, 22). Phosphorylation of p38 MAP kinase is also involved in integrin activation. Calcium and diacylglycerol-regulated guanine nucleotide exchange factor-1 and RAS-related protein 1a (Rap1a) are downstream of Syk (23). PSGL-1 polarization is cytoskeleton-dependent, and the N-terminal ITAM of moesin can create a “bridge” between the cytoplasmic domain of PSGL-1 and Syk (24–26). Active Syk propagates activation signals to downstream mediators that extend β_2_ integrins (8, 10, 27). The integrity of lipid rafts is also necessary for β_2_-integrin activation through the PSGL-1 axis (24). As a necessary leading event of β_2_ integrins in leukocytes, cellular calcium ion (Ca^2+^) signaling of neutrophils on immobilized P-selectin requires a stimulus time of ~75 s under a wall shear stress of 0.2 Pa and may be *via* the MAPK pathway, but not in a lipid raft-dependent manner (28). This timescale for a “Ca^2+^ burst” >1 min reveals the existence of slow P-selectin-induced β_2_-integrin activation with a timescale of a few minutes. This discordance in the function of lipid rafts is incompletely understood. Meanwhile, disruption of the actin cytoskeleton is highly suppressive to β_2_-integrin activation through immobilized chemokine signaling (18). Ezrin/radixin/moesin (ERM) connects the cytoplasmic domains of PSGL-1 and CD44 with the cytoskeleton (25, 26, 29, 30). Talin-1 (a member of a four-point-one, ezrin, radixin, moesin (FERM)-domain protein family) binds the cytoplasmic tail of an integrin and recruits cytoskeletal and signaling proteins to activate integrins synergistically (6). Hence, β_2_-integrin activation could occur *via* a non-MAPK pathway from PSGL-1, ERM, or talin-1 to the cytoplasmic tail of integrins.

In a hemodynamic microenvironment, the fluid load on adhered cells serves as a regulator for activation of β_2_-integrin signaling. Fluid shear stress regulates the rolling and stopping of rolling through force-dependent bond dissociation of PSGL-1 from E- or P-selectin (31, 32), which causes a duration adjustment in activation of β_2_-integrin signaling *via* the PSGL-1/selectin axis. A force-induced increase in the stopping time of cells may suggest force-enhanced maturation of β_2_ integrins (32) if PSGL-1 dissociation from selectin obeys the “catch bond” mechanism (31, 33). In comparison with rolling neutrophils, suspended neutrophils in the absence of shear stress produce few and weak Ca^2+^ signals and show minimal upregulation of β_2_-integrin affinity (28, 34). However, the force-accelerated Ca^2+^ bursts of neutrophils on immobilized P-selectin (28) suggest that a mechanical stimulus is a dominant mediator for β_2_-integrin activation and its spatiotemporal characteristics (even though the knowledge of selectin-induced β_2_-integrin activation and its spatiotemporal characteristics is very poor).

Here, we examined cell tethering, Ca^2+^ bursts, and activation of β_2_-integrin signaling of human neutrophils on functionalized substrates through a parallel plate flow chamber (PPFC) experiment using immunofluorescence. We discovered two signaling pathways for β_2_-integrin activation of neutrophils on immobilized P-selectin in flow: a fast (but local) pathway and a slow (but global) pathway. Engagement with ICAM-1 or GPIbα mediated the transition from local integrin activation to global integrin activation. This action was followed by accelerated integrin activation over the entire cell surface. The flow load triggered Ca^2+^ bursts and activation of β_2_-integrin signaling of neutrophils on P-selectin in flow. Lipid rafts, moesin, actin, and talin-1 were involved in fast signaling, but Syk, lipid rafts, and moesin were involved in slow signaling. These results provide novel insights into β_2_-integrin activation, a key early event in the recruitment of flowing leukocytes to inflammatory sites of vascular surfaces.

## Results

### Force triggers P-selectin-induced extension of β_2_ integrins over the entire neutrophil surface in an ICAM-1- or GPIbα-enhanced manner

First, we examined β_2_-integrin activation of firmly adhered human neutrophils on immobilized P-selectin (10 μg/mL) with or without co-immobilized ICAM-1 (5 μg/mL) or GPIbα (40 μg/mL). This P-selectin density of 10 μg/mL was selected to support specific firm adhesion of neutrophils on substrates (**Supplementary Figure S1**) (28). The firmly adhered cells were loaded with a wall shear stress (WSS) of 0.2 dyne/cm^2^ for different times (0.0, 0.5, 1.0, 2.0, and 3.0 min) and KIM127 (Alexa Fluor 594-labeled β_2_-subunit extension reporter antibody) was used to identify the β_2_-integrin extension on cells. Alexa Fluor 488-labeled IBL-6/2 and M1/70 (CD11a (α_L_-subunit) and CD11b (α_M_-subunit) recognition antibodies) were used to recognize the β_2_-integrin family members LFA-1 and Mac-1, respectively.

**Figure 1:**
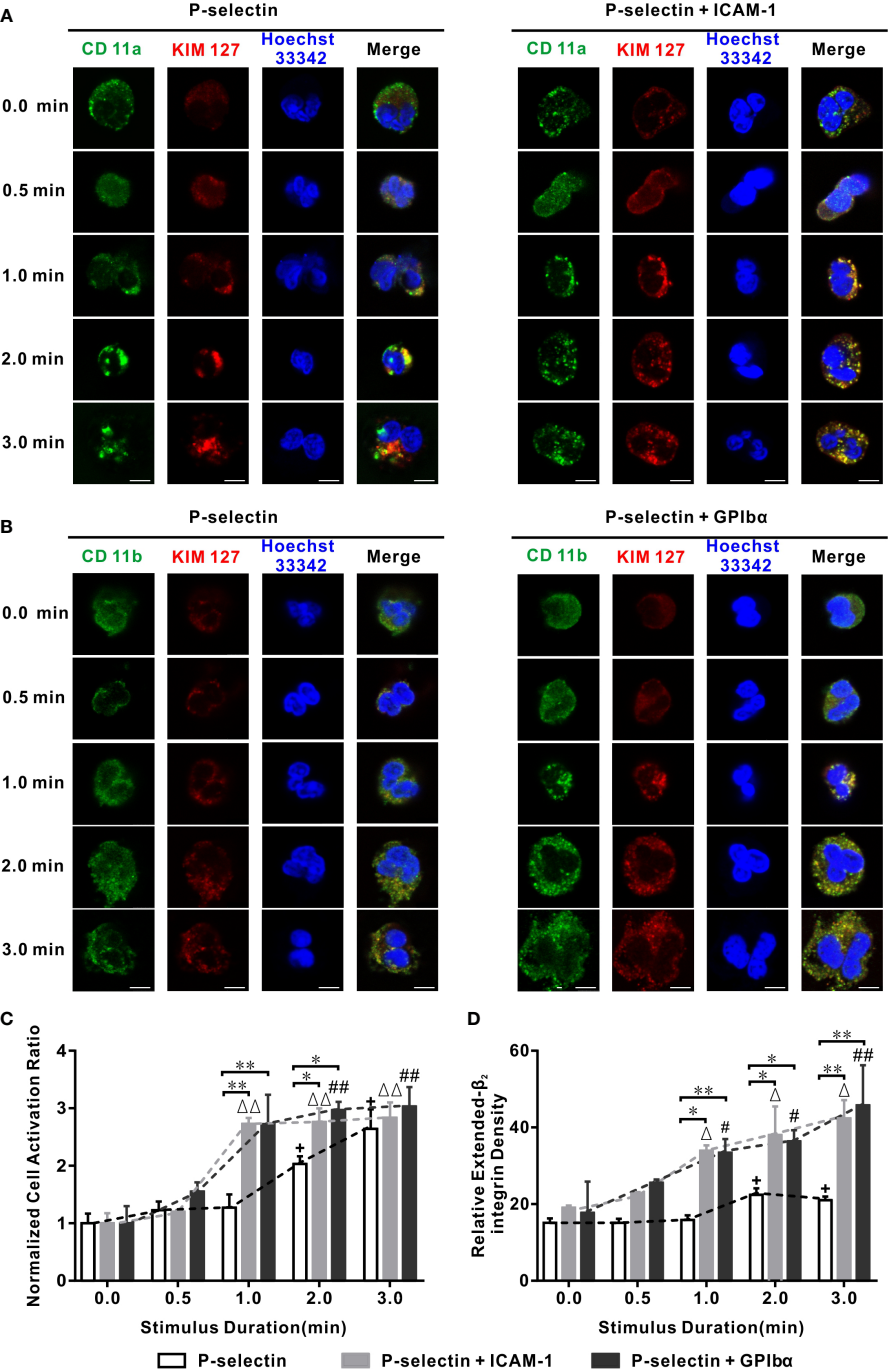
Variation of cell activation ratio, extended β_2_ integrin pattern, and intensity versus stimulus time in flow. Wall fluid shear stress of 0.2 dyne/cm^2^ was loaded to neutrophils on P-selectin plus ICAM-1, GPIbα, or nothing for different loading times. **(A, B)** Representative patterns and their merged images of Hoechst 33342-stained nuclei (blue) and β_2_ integrin bound with anti-CD11a Ab (green), anti-CD11b Ab (green), and KIM127(red). The scale bars = 5 μm for each image. **(C, D)** Plots of normalized activation ratio and relative extended integrin density of firmly adhered cells versus stimulus time. In **(C, D)**, the data were represented the mean ± SEM from at least three independent experiments, and every experiment contained 10 different visual sights. Statistical significance was analyzed by two-way ANOVA for multiple comparisons, the significant level of difference was shown by *P*-value, #**, Δ** or **+** for *P* < 0.05 and **##, ΔΔ** or **++** for *P* < 0.001 from static condition, * *P* < 0.05 and ** *P* < 0.001 from P-selectin column.

The KIM127-ligated β_2_ integrin phases (red) in the absence of ICAM-1 or GPIbα that formed over the cells shared topologic structures with anti-CD11a or CD11b antibody-ligated β_2_-integrin phases (green), respectively. These phases were weak initially and then became intensively dose-dense as the stimulus duration increased. Engagement with ICAM-1 or GPIbα enhanced formation of KIM127-ligated β_2_-integrin phases (red), especially under a fluid load for 0.5 min (**Figures 1A, B**). This observation revealed that activation of a P-selectin-induced β_2_-integrin process occurred on the entire cell surface. It also suggested that a shift from a bent conformation to an extended (red) conformation emerged in integrins over the entire cell surface that involved ICAM-1- or GPIbα-mediated enhancement which was weak initially but which strengthened as the loading time increased.

We wished to quantify the temporal characteristics of extension of β_2_-integrin conformations. We used two parameters: (i) relative density of extended-β_2_ integrins (KIM127-related fluorescence intensity of the cell over that of the background minus one); (ii) cell activation ratio (proportion of β_2_ integrin extension-positive cells in all firmly adherent cells), which was normalized through dividing it by its value in the absence of a mechanical load. Plots of the normalized cell activation ratio and relative density of extended-β_2_ integrins against the stimulus time at a wall shear stress of 0.2 dyne/cm^2^ (**Figures 1C, D**) showed that a stimulus time ≥30 s was required for the extension (or moderate activation) of β_2_ integrins over cells on P-selectin irrespective of whether ICAM-1 and GPIbα were present. The normalized cell activation ratio increased with the stimulus time, as did the relative density of extended-β_2_ integrins, for cells on P-selectin with or without ICAM-1 or GPIbα (**Figures 1C, D**). In the absence of ICAM-1 or GPIbα, the normalized cell activation ratio and relative density of extended-β_2_ integrins remained at their respective (almost static) low levels of 1.0 and 16 initially, then climbed rapidly to *plateaus* of 2.1 and 24, and the phase switch occurred at a stimulus-time threshold of 1–2 min, whereas engagement with ICAM-1 or GPIbα halved these stimulus-time thresholds and heightened the *plateaus* considerably (**Figures 1C, D**; **Supplementary Figure S2**). In contrast, LFA-1 activation of rolling cells on E-selectin reached a stable high level under a fluid flow stimulus of ~50 s (10), whereas the transition from rolling to arrest for neutrophils on substrates (P-selectin/ICAM-1/IL-8) occurred within 30 s (7). These similar time scales revealed the temporal characteristics of β_2_-integrin activation of neutrophils that rolled-on or adhered to substrates.

**Figure 2:**
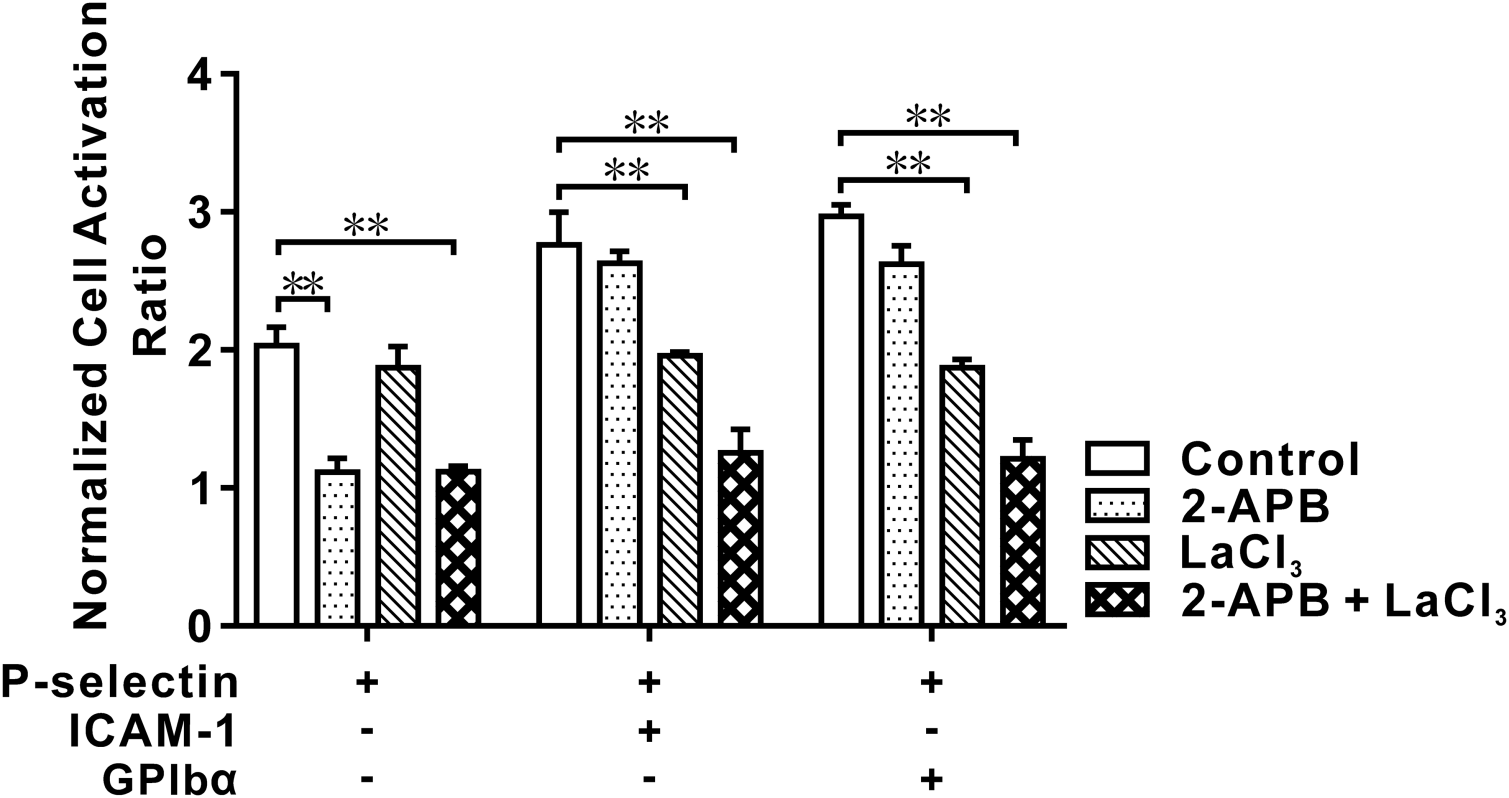
Effect of blocking IP_3_ and/or membrane calcium channel on cell activation in flow. Neutrophils were pretreated with IP_3_ receptor restrainer 2-APB, membrane calcium channel blocker LaCl_3,_ and nothing (for control) before perfusing into the flow chamber under wall fluid shear stress of 0.2 dyne/cm^2^ for 2.0 min. The data showed an inhibitory effect of blocking IP_3_ and/or membrane calcium channel on activation of cells on P-selection plus ICAM-1, GPIbα or nothing, and represented the mean ± SEM from three independent experiments, and every experiment contained 10 different visual sights. Statistical significance was analyzed by two-way ANOVA for multiple comparisons. The significant level of difference was shown by *P*-value, ** *P* < 0.001. The treatments of functionalized substrates had a significant effect on the normalized cell activation ratio.

The cellular Ca^2+^ response is responsible for chemokine- and selectin-mediated β_2_-integrin activation (28, 34). We examined which type of Ca^2+^ signaling was involved in global integrin activation. For cells on P-selectin, blockade of intracellular Ca^2+^ release by 2-aminoethoxydiphenyl borate (2-APB) reduced the cell activation ratio significantly in the absence of ICAM-1 or GPIbα, but decreased it slightly in the presence of ICAM-1 or GPIbα, whereas blockade of extracellular Ca^2+^ influx by LaCI_3_ had the opposite effect. Also, blockade of extracellular Ca^2+^ influx and intracellular Ca^2+^ release reduced the cell activation ratio more significantly in the presence of ICAM-1 and GPIbα, but did not do so in the absence of ICAM-1 and GPIbα (**Figure 2**). These data suggested that global integrin activation was relevant only to Ca^2+^ signaling through intracellular Ca^2+^ release for cells on P-selectin alone. Interestingly, engagements with ICAM-1 or GPIbα triggered another type of Ca^2+^ signaling through extracellular Ca^2+^ influx, which regulated global integrin activation together with intracellular Ca^2+^ release.

These data suggested that fluid shear stress triggered activation of β_2_ integrins over neutrophils on immobilized P-selectin with or without ICAM-1 or GPIbα. Before global integrin activation, local integrin activation should occur rapidly at the cell-contact area. The freshly activated integrin binds with its ligands (ICAM-1 or GPIbα) first and then triggers Ca^2+^ signaling through extracellular Ca^2+^ influx to quicken extension of integrins over the entire cell surface (**Figures 1**, **2**).

### ICAM-1 and GPIbα accelerate P-selectin-mediated Ca^2+^ bursts in neutrophils *via* extracellular Ca^2+^ influx in a force-dependent manner

**Figure 3:**
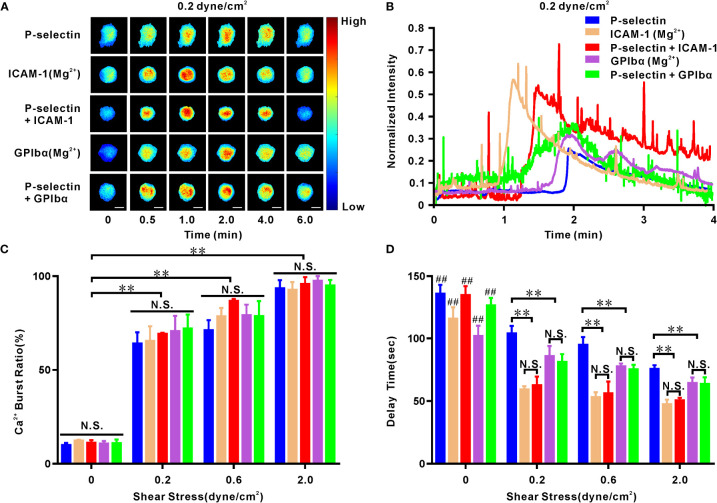
Cellular calcium responding of human neutrophils to mechano-chemical stimuli. Cells firmly adhered to ICAM-1, GPIbα or P-selectin (combined with ICAM-1, GPIbα or nothing) under various wall shear stresses for different times. On ICAM-1 or GPIbα alone, the cells were pretreated with Mg^2+^ to make integrin extended. **(A)** Representative fluorescence images of cellular calcium bursting under wall shear stress of 0.2 dyne/cm^2^ at different loading times. Scale bar = 5 μm. **(B)** Representative time-courses of normalized fluorescence intensity over entire cell surface within loading time of 4 minutes at wall shear stress of 0.2 dyne/cm^2^. Plots of calcium burst ratio **(C)** and delay time **(D)** within load time of 6 min versus wall shear stress. In **(C, D)**, the data represented the mean ± SEM from at least 20 cells and three independent experiments. Statistical significance was analyzed by two-way ANOVA for multiple comparisons. The significant level of difference was shown by *P*-value, N.S. represented for no significant difference, and ** for *P* < 0.001 from static condition **(C)** or P-selectin **(D)**. The significant level of difference between the cells at static condition and cells in flow (0.2, 0.6, or 2.0 dyne/cm^2^) on the same substrate is shown by *P*-value, ## for *P* < 0.001. Calcium burst ratio was positively correlated with wall shear stress, because their Pearson’s correlation coefficient r was within range from 0.64 to 0.77 with *P* < 0.01 for cells on ICAM-1, GPIbα, P-selectin, P-selectin plus ICAM-1 or GPIbα; but calcium burst delay time was negatively correlated with wall shear stress, because of their Pearson’s correlation coefficient r within range from -0.48 to -0.35 with *P* < 0.001 for cells on each substrate mentioned above.

Early extension of integrins on the cell-contact area should be reflected by the cellular Ca^2+^ response in the presence of ICAM-1 and/or GPIbα because moderately activated integrins should initiate Ca^2+^ signaling through extracellular Ca^2+^ influx by binding to ICAM-1 or GPIbα (**Figure 3**). We undertook PPFC experiments under various values of wall shear stress in real-time to examine Ca^2+^ bursts of firmly adherent neutrophils on immobilized P-selectin, ICAM-1, or GPIbα alone as well as immobilized P-selectin plus co-immobilized ICAM-1 or GPIbα (**Supplementary Movie 1**). Cells were pretreated with Mg^2+^ to extend β_2_ integrins before perfusion over substrates coated with ICAM-1 alone or GPIbα alone.

**Figure 4:**
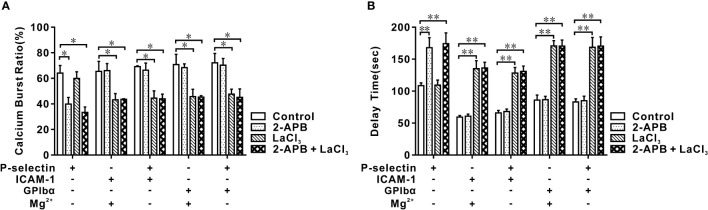
Effects of blocking IP_3_ and membrane calcium channel on calcium bursting of human neutrophils in flow. Cells were pretreated with 2-APB, LaCl_3,_ and nothing (control), and perfused over PPFC for 7 minutes at wall shear stress of 0.2 dyne/cm^2^. Five different substrates were coated with ICAM-1, GPIbα, and P-selectin with or without ICAM-1 or GPIbα. Calcium burst ratio **(A)** and delay time **(B)** of calcium signaling of cells on these substrates. Blockage of IP_3_ inhibited P-selectin-induced cytosolic calcium release instead of integrin-mediated extracellular calcium influx, and blockage of membrane calcium channel did inversely. In **(A, B)**, the data represented the mean ± SEM from at least 20 cells from three independent experiments. Statistical significance was analyzed by two-way ANOVA for multiple comparisons. The significant level of difference was shown by *P*-value, * *P* < 0.05 and ** *P* < 0.001. The treatments of functionalized substrates did not have a significant effect on calcium burst ratio **(A)** but had a significant variation on delay time **(B)**.

Cellular fluorescence images (**Figures 3A, B**) revealed the diverse Ca^2+^ responses of neutrophils (on P-selectin alone and with ICAM-1 or GPIbα) and Mg^2+^-treated-neutrophils (on ICAM-1 alone or GPIbα alone) under a wall shear stress of 0.2 dyne/cm^2^ for loading times of 0.0, 0.5, 1.0, 2.0, 4.0, and 6.0 min. The cellular Ca^2+^ burst ratio (number of Ca^2+^ burst-positive cells over the total number of firmly adherent cells) remained at a low (but stable) level in a static state, increased steeply first and then moderately with wall shear stress, but showed no significant difference within various substrates at each given wall shear stress (**Figure 3C**). These data suggested that force triggered adhesion molecule-mediated cellular Ca^2+^ bursts. The normalized fluorescence intensity of cells (instantaneous fluorescence intensity over the initial fluorescence intensity) under a wall shear stress of 0.2 dyne/cm^2^ (**Figure 3B**) was maintained almost constantly initially, then climbed rapidly to a peak and, finally, declined gradually to its initial level as the loading time increased. However, it remained ~1 within an observation period of 200 s at the static state, as shown in studies for the Ca^2+^ bursts of cells on P-selectin only (28) or E-selectin in the presence of perfused chemokines (34). However, peak shifts were present in these similar Ca^2+^-burst patterns of neutrophils on differentially treated substrates (**Figure 3B**), which were calibrated by a latent period or delay time (the duration from firm adhesion to the Ca^2+^ burst of the cell) (**Figure 3D**). Under a given wall shear stress (0.2, 0.6, or 2.0 dyne/cm^2^), the delay times of Ca^2+^ bursts for Mg^2+^-treated neutrophils on ICAM-1 alone and GPIbα alone were almost identical to those for neutrophils on P-selectin with ICAM-1 or GPIbα, respectively, but significantly shorter than those on P-selectin only (**Figure 3D**). Hence, engagement with GPIbα or ICAM-1 quickened Ca^2+^ bursts of neutrophils on P-selectin under WSS of 0.2, 0.6, or 2.0 dyne/cm^2^. This phenomenon of GPIbα or ICAM-1 engagement-mediated acceleration of cellular Ca^2+^ bursts (**Figure 3D**) provides further evidence for fast P-selectin-induced activation of β_2_ integrins on cell-contact areas under WSS of 0.2, 0.6, or 2.0 dyne/cm^2^. This is because engagement with GPIbα or ICAM-1 required local activation of LFA-1 and Mac-1 on the cell-contact area, respectively, and the almost identical delay times of Ca^2+^ bursts for Mg^+^-treated neutrophils (on ICAM-1 alone or GPIbα alone) and neutrophils (on P-selectin plus ICAM-1 or GPIbα) (**Figure 3D**) revealed very fast activation of β_2_-integrin signaling. In addition, the delay time of Ca^2+^ bursts decreased rapidly with an increase in wall shear stress (<0.2 dyne/cm^2^) initially and then remained at a constant (but lower) level when wall shear stress ≥0.2 dyne/cm^2^ for neutrophils on ICAM-1 alone or with P-selectin, whereas wall shear stress shortened the delay time of Ca^2+^ bursts slightly for neutrophils on GPIbα, P-selectin alone, or in combination with each other (**Figure 3D**). However, the delay times of Ca^2+^ bursts of ~1 min and ~1.4 min were longer than the integrin activation-required loading times of ~1 min for cells on P-selectin with ICAM-1 or GPIbα, respectively (**Figures 1C, D**, **3D**, **4B**). Hence, non-real-time measurement underestimated the integrin activation-required loading time.

Whether Ca^2+^ bursts arose from extracellular Ca^2+^ influx or intracellular Ca^2+^ release for different substrates is not known. Thus, we preincubated neutrophils with 2-APB and/or lanthium trichloride (LaCl_3_) before perfusing a cell suspension into the flow chamber. Under a wall shear stress of 0.2 dyne/cm^2^ and a loading time of 6 min, 2-APB pretreatment reduced the Ca^2+^ burst ratio and prolonged the delay time of Ca^2+^ bursts prominently for cells on P-selectin alone, but not upon LaCl_3_-pretreatment. In contrast, LaCl_3_ pretreatment reduced the Ca^2+^ burst ratio and led to a significant increase in the delay time for Ca^2+^ bursts on cells (on P-selectin plus ICAM-1 or GPIbα) and Mg^2+^-treated cells (on ICAM-1 or GPIbα), but not upon 2-APB pretreatment (**Figures 4A, B**). Hence, Ca^2+^ bursts were derived dominantly from P-selectin/PSGL-1 axis-mediated cytosolic Ca^2+^ release or integrin/ICAM-1 (or GPIbα) axis-mediated extracellular Ca^2+^ influx. Also, the Ca^2+^ burst ratios and delay times for Ca^2+^ burst cells on P-selectin plus ICAM-1 or GPIbα had a negligible difference from those of Mg^2+^-treated cells on ICAM-1 or GPIbα. These data suggested that integrins on the cell-contact area were activated rapidly by P-selectin initially and then contributed to meditating extracellular Ca^2+^ influx through binding with ICAM-1 and/or GPIbα under WSS of 0.2, 0.6, or 2.0 dyne/cm^2^.

### Engagement with ICAM-1 and GPIbα prolongs the biphasic force-dependent tether lifetime of neutrophils on P-selectin in flows

We wished to quantify the temporal characteristics of local integrin activation through the PSGL-1 axis. We examined the transient tethering events (stopping between two consecutive movements) of neutrophils on substrates coated with P-selectin (30 ng/mL) alone or with either ICAM-1 (500 ng/mL) or GPIbα (20 μg/mL) under fluid wall shear stress from 0.1 dyne/cm^2^ to 0.45 dyne/cm^2^ (**Supplementary Movie 2**), and collected the tether lifetimes or stopping times of these tethering events (**Supplementary Figure S3**). These tethering events were specific for P-selectins because they were eliminated through incubation with a P-selectin-blocking antibody (KPL-1) irrespective of whether ICAM-1 and GPIbα were present (**Supplementary Figure S4A**). Such densities of P-selectin, ICAM-1, and GPIbα could ensure that some cells near the bottom of the flow chamber stopped briefly, but did not support rolling or skipping on the flow-chamber bottom (31). Hence, most tethering events could be mediated by a single adhesive molecular bond formation in flow, based on the possibility model and Monte Carlo simulation (35).

**Figure 5:**
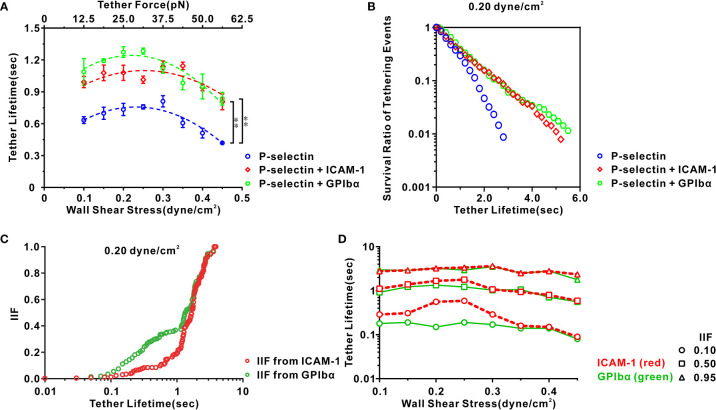
Characteristics of transient tether events of neutrophils on three functionalized substrates under various shear stresses. **(A)** Variation of tether lifetime versus wall shear stress for cells on P-selectin with ICAM-1 (red), GPIbα (green), or nothing (blue). Their corresponding dash lines represented binomial curve fitting. Plots of **(B)** survival ratio and **(C)** integrin-involved fraction (IIF) of tethering events versus tether lifetime for cells on P-selectin in presence of ICAM-1 (red), GPIbα (green) or nothing (blue) at wall shear stress of 0.20 dyne/cm^2^. IIF represented a possibility that cell remained tethered through binding of integrin with ICAM-1 or GPIbα instead of P-selectin with PSGL-1 at a given tether lifetime and was evaluated by (*P*_E_-*P*)/*P*_E_, where *P*_E_ and *P* were the survival ratios of tethering events in the existence and absence of ICAM-1 or GPIbα, respectively. **(D)** Plots of tether lifetime versus wall shear stress at IIF = 0.10, 0.50, and 0.95 in the existence of ICAM-1 (red) and GPIbα (green). The tether lifetimes at IIF = 0.10, 0.50, and 0.95 reflected requirements of mechanical load time for initial, moderate, and sufficient activation of integrin nearby ligated PSGL-1, respectively. In **(A)**, the data represented the mean ± SEM from at least 250 tether events in at least three independent experiments. Statistical significance was analyzed by two-way ANOVA for multiple comparisons. The significant level of difference was shown by *P*-value, ** for *P* < 0.001.

Plots of the mean tether lifetime *versus* wall shear stress (**Figure 5A**) showed that the tether lifetime lengthened first and then shortened as the wall shear stress increased from 0.1 dyne/cm^2^ to 0.45 dyne/cm^2^ for cells on immobilized P-selectin combined with ICAM-1, GPIbα, or no agent, as reported previously for HL60 cells on P-selectin alone (31). Importantly, engagement with ICAM-1 or GPIbα lengthened the mean tether lifetime at a wall shear stress from 0.1 dyne/cm^2^ to 0.45 dyne/cm^2^. Blockade of LFA-1 and Mac-1 on cells by anti-LFA-1 antibodies and anti-Mac-1 antibodies removed the effects of ICAM-1 and GPIbα on the mean tether lifetime at a wall shear stress of 0.45 dyne/cm^2^ (**Supplementary Figure S4B**). This result suggested that once a flowing cell was tethered to P-selectin, β_2_ integrins associated with the P-selectin/PSGL-1 axis might become extended within a mean cell stopping time of 0.4–0.8 s and enhance the tethering event through binding of freshly activated integrin to ICAM-1 or GPIbα under flow (**Figure 5A**; **Supplementary Figure S4B**). The distribution of patterns of the tether lifetime for cells on P-selectin with ICAM-1 or GPIbα at a wall shear stress from 0.1 dyne/cm^2^ to 0.45 dyne/cm^2^ showed two peaks. One peak occurred within ~0.7 s in accordance with a Gauss-fitted peak of the distribution pattern of the tether lifetime for neutrophils on immobilized P-selectin alone. The other occurred at ~1 s (**Supplementary Figures S5A, B**). These data provided further support for the notion that β_2_-integrin activation is *via* P-selectin-mediated cell-stopping events.

We wished to reveal more deeply the spatiotemporal features of local integrin activation under WSS of 0.1, 0.15, 0.2, 0.25, 0.3, 0.35, 0.4, or 2.0 dyne/cm^2^. The survival ratio and integrin-involved fraction (IIF) of tethering events at a given time *t* were introduced and evaluated using *N*/*N*_T_ and (*P*_E −_ *P*)/*P*_E_, where *N*_T_ denotes the total number of events and *N* is the number of the events with a lifetime ≥*t*, and *P*_E_ and *P* are the survival ratios of tethering events in the presence and absence of ICAM-1 or GPIbα, respectively. The IIF represents the possibility that a cell remained tethered by binding integrins to ICAM-1 or GPIbα instead of P-selectin to PSGL-1 at a given tether lifetime. Plots of the survival ratio *versus* tether lifetime (**Figure 5B**; **Supplementary Figure S6A**) showed that engagement with ICAM-1 or GPIbα led to a significant increase in the survival ratio of a tethering event at a wall shear stress of 0.1–0.45 dyne/cm^2^. Plots of the IIF *versus* tether lifetime (**Figure 5C**; **Supplementary Figure S6B**) showed the IIF to be at a latency stage of ~0.1 initially and then increased steeply to 1 as the tether lifetime increased from 0.1 s to 2 s for cells on P-selectin in the presence of ICAM-1 or GPIbα at a wall shear stress of 0.20 dyne/cm^2^. These data suggested local P-selectin-mediated integrin activation that occurred at the P-selectin/PSGL-1 axis within sub-seconds. Under a wall shear stress from 0.1 dyne/cm^2^ to 0.45 dyne/cm^2^, the tether event had a lifetime from 0.08 s to 0.19 s, 0.56 s to 1.34 s, and 1.78 s to 3.52 s once the IIF from GPIbα reached 0.10 s, 0.50 s, and 0.95 s, respectively. However, an increment in the tether lifetime at these values of the IIF from ICAM-1 was noted (**Figure 5D**), indicating that steady mechanical stimuli of at most 0.2, 1.4 and 3.5 seconds through the P-selectin/PSGL-1 axis were required for initial, moderate, and complete integrin activation, respectively. These results suggested that local P-selectin-induced integrin activation was very fast (<0.2 s) once the cell was tethered to P-selectin, and would become significant within a tether lifetime of a few seconds.

To ascertain this conformational change of β_2_ integrins of neutrophils on immobilized P-selectin under wall shear stress, we used the antibodies KIM127 and mAb24 to recognize β_2_ integrins with moderate and high affinity, respectively (36, 37). At a wall shear stress of 0.2 dyne/cm^2^, activated β_2_ integrins were more likely to be in an extended state instead of a high-affinity state (**Supplementary Figure S7**), similar to LFA-1 activation of lymphocytes on immobilized chemokines (18).

### Moesin-actin-talin might be involved in β_2_-integrin activation at global and local levels

Cellular Ca^2+^ bursts are early and necessary events in activation of global β_2_-integrin signaling of neutrophils on immobilized P-selectin in flow (34), but did not occur in the local integrin activation (**Figures 2**–**5**). These data suggested at least two signaling pathways were involved in β_2_-integrin activation. To test this hypothesis, we carried out PPFC experiments to examine the effects of various molecular inhibitors on the Ca^2+^ bursts and tethering events of neutrophils. Neutrophils were tethered to or adhered firmly on immobilized P-selectin with ICAM-1, GPIbα, or no substrate. Neutrophils were pretreated with piceatannol (inhibitor of Syk kinase), methyl-β-cyclodextrin (MβCD; disruptor of lipid rafts), 3,4-methylenedioxy-beta-nitrostyrene (MNS; indirect inhibitor of talin), cytochalasin B (actin disruptor), staurosporine (moesin inhibitor), dimethyl sulfoxide (DMSO) or no agent (control), under a wall shear stress of 0.2 dyne/cm^2^.

**Figure 6:**
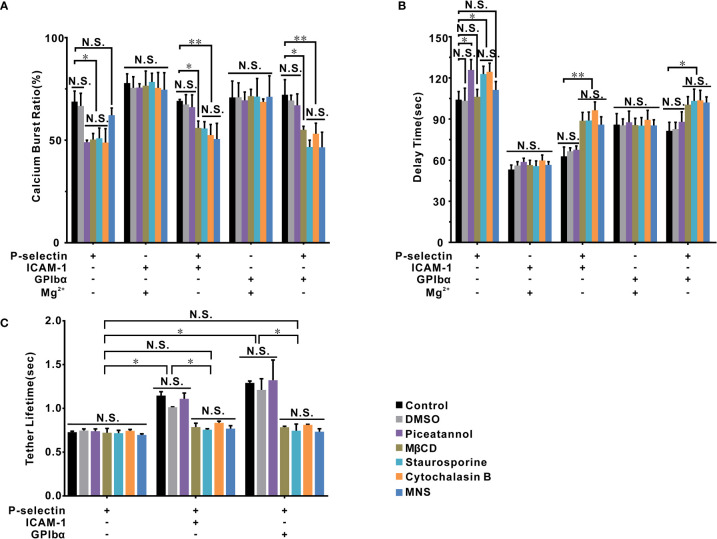
The signal molecules involved in β_2_ integrin activation of cells on P-selectin. Calcium burst ratio **(A)** and delay time **(B)** of calcium signaling of firmly adhered human neutrophils, which were pretreated with or without piceatannol, MβCD, staurosporine, cytochalasin B, and MNS, at wall shear stress of 0.2 dyne/cm^2^. Cells were pretreated with Mg^2+^ to activate LFA-1 and Mac-1 if substrates were coated only with ICAM-1 or GPIbα. **(C)** The mean tether lifetime of cells on the substrates shown on the panel was measured at wall shear stress of 0.2 dyne/cm^2^ after cells were treated with different inhibitors. In **(A, B)**, the data represent the mean ± SEM from at least 20 cells in three independent experiments. In **(C)**, at least 191 samples were measured under each condition. The data represented mean ± SEM in at least three independent experiments. Control (the cells treated with nothing) and DMSO groups were used as parallel control groups. Statistical significance was analyzed by two-way ANOVA for multiple comparisons. The significant level of difference is shown by *P*-value, * *P* < 0.05, ** *P* < 0.001, N.S. for no significant difference.

The tether lifetime (~1.25s) of cells on P-selectin combined with ICAM-1 or GPIbα was shortened (~0.7s) by depolymerizing actin with cytochalasin B, disrupting lipid rafts with MβCD, blocking moesin with staurosporine, and inhibiting talin with MNS (**Figure 6**). Blockade of Syk with piceatannol did not affect the tether lifetime, which suggested a local (but rapid) P-selectin/PSGL-1-induced activation of β_2_-integrin signaling *via* signal transducers such as lipid rafts, moesin, actin and talin. None of these treatments could regulate the tether lifetime (~0.7s) for cells on P-selectin alone. Hence, binding of PSGL-1 to P-selection was responsible for most of the tether events (**Figure 6C**). Studies have demonstrated that PSGL-1 can associate with lipid rafts (38), moesin is a linker between actin and PSGL-1 (26, 39), the cytoskeleton actin can connect to moesin and talin (40, 41), and talin is an important adaptor to link integrins and cytoskeletal actin (42, 43).

These signal transducers could also be involved in P-selectin-induced Ca^2+^ bursts. Treatment with MβCD, staurosporine, or cytochalasin B significantly reduced the cellular Ca^2+^ burst ratio in the absence and presence of ICAM-1 or GPIbα, as did piceatannol treatment in the absence of ICAM-1 or GPIbα, and MNS treatment only in the presence of ICAM-1 or GPIbα (**Figure 6A**). These results suggested two P-selectin-induced Ca^2+^ signaling pathways dependent on Syk and talin in the absence and presence of ICAM-1 or GPIbα, respectively, which shared the signal transducers actin, moesin, and lipid rafts. Besides, in the absence of ICAM-1 and GPIbα, treatments with piceatannol, staurosporine, and cytochalasin B increased the delay time of Ca^2+^ bursts (**Figure 6B**), but treatments with MNS or MβCD did not. In the presence of ICAM-1 or GPIbα, treatments with MNS, MβCD, staurosporine, and cytochalasin B lengthened the delay time of Ca^2+^ bursts, but piceatannol treatment did not (**Figure 6B**). The five signaling molecules mentioned above were not involved in Ca^2+^ signaling through an extended (or Mg^2+^-treated) β_2_-integrin axis because their treatment had no effects on the Ca^2+^ burst ratio or delay time of Ca^2+^ bursts (**Figures 6A, B**).

## Discussion

In recruitment of flowing leukocytes to inflammatory vascular sites, early cellular and molecular events include cell tethering, cell rolling, firm adhesion of cells, and integrin activation (12). The spatiotemporal characteristics of integrin activation regulate these cellular events, but are incompletely understood.

**Figure 7:**
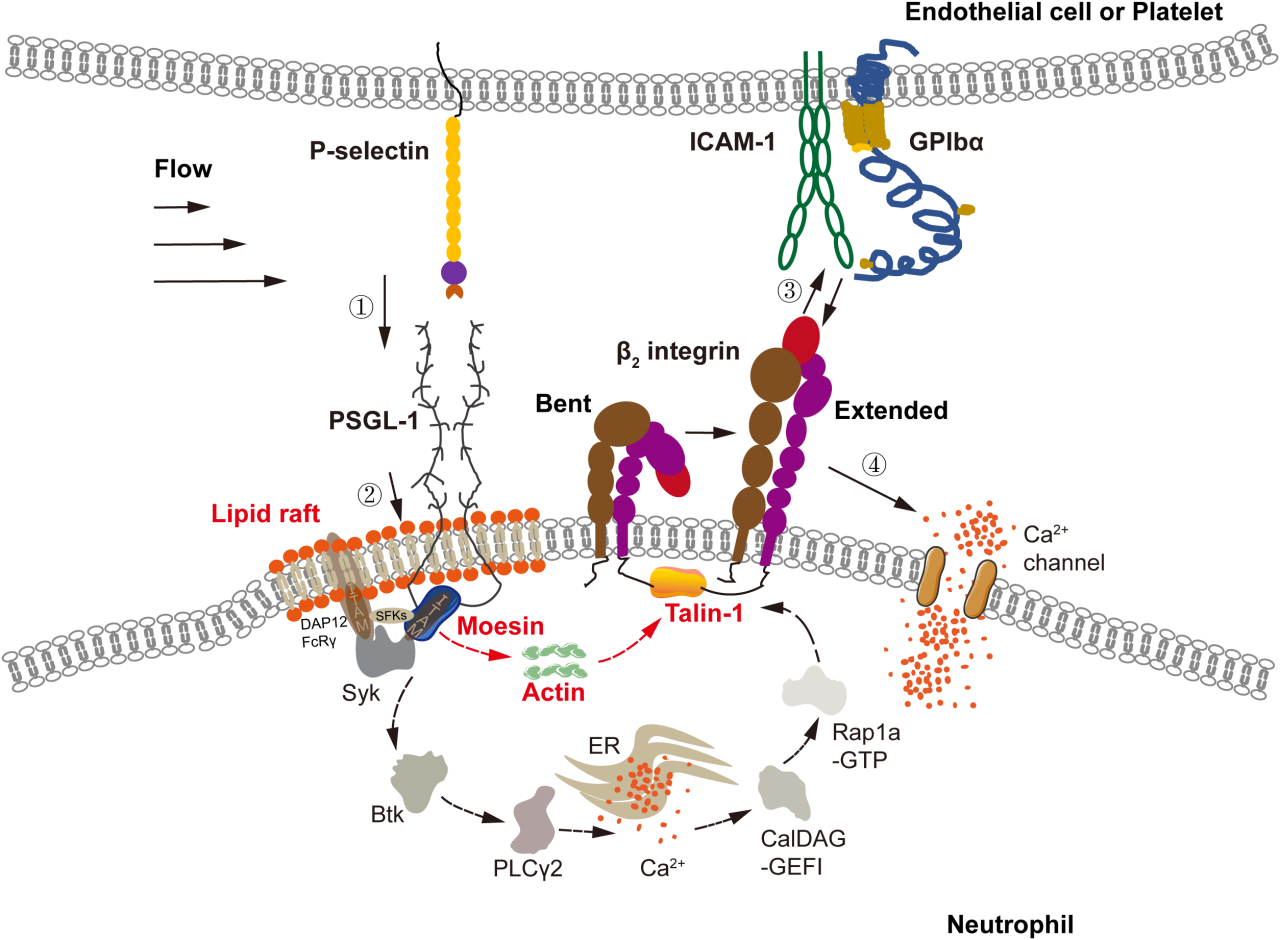
Model for P-selectin-mediated activation *via* a non-conventional signal pathway. In flow, P-selectin on activated endothelial cells or platelets engaged with PSGL-1 on neutrophils to initiate integrin activation signaling *via* MAPK pathway, which includes signaling components, such as lipid rafts, three SFKs (Hck, Lyn, Fgr), ITAM adaptors (DAP12 and FcRγ), Syk, Btk, PLCγ2, CalDAG-GEFI, Rap 1a-GTP, and talin-1, and requires intracellular Ca^2+^ release instead of extracellular Ca^2+^ influx, as shown in previous work (21–24, 27, 44–46). Through the MAPK pathway, P-selectin-mediated extending of integrin over entire cell surface has a timescale of minutes. And, integrin expanding *via* non-MAPK signal firstly ensues from P-selectin/PSGL-1 signaling of sub-seconds and occurs at the ligated PSGL-1 neighborhood. Liquid raft, moesin, actin, and talin-1 were involved in the non-MAPK signaling with a subsecond timescale. Binding of the freshly extended β_2_ integrin near ligated PSGL-1 to ICAM-1 on endothelial cell or GPIbα on platelet initiates calcium signaling *via* extracellular Ca^2+^ influx instead of intracellular Ca^2+^ release, mediating subsequent extending of integrin over entire cell surface within 1-2 minutes. The calcium signaling *via* either extracellular Ca^2+^ influx or intracellular Ca^2+^ release has a timescale of a few minutes, like the global integrin activation.

We discovered that a flowing load triggered β_2_-integrin activation of neutrophils on immobilized P-selectin *via* MAPK and non-MAPK signaling (**Figure 7**). The MAPK pathway (6, 15, 44) was involved in activation of integrins on the entire cell surface for a few minutes in a Syk-dependent manner. With respect to the non-MAPK pathway, activation of signal transduction comprised two stages. The first stage was involved in activation of integrins adjacent to ligated PSGL-1 within sub-seconds. The second stage was related to subsequent engagement with ICAM-1 or GPIbα which induced activation of integrins over the entire cell surface within a few minutes. Different from MAPK signaling, ICAM-1 or GPIbα engagement-induced activation of β_2_ integrins required extracellular Ca^2+^ influx instead of cytosolic Ca^2+^ release (**Figure 7**).

Our data suggest transition from a local (but rapid) to a global (but slow) mode of integrin activation *via* non-MAPK signaling (**Figure 7**). In non-MAPK signaling cascades, integrin activation occurred rapidly (within sub-seconds) near ligated PSGL-1 through signaling from PSGL-1, moesin, actin, and talin-1 to β_2_ integrins initially. Then, the freshly activated β_2_ integrins were bound with their ligands to form “crosstalk” between neutrophils and endotheliocytes (1) or platelets (47). Also, by “flipping the switch” of the transition mode of β_2_-integrin activation, this cell–cell crosstalk initiated subsequent Ca^2+^-dependent integrin activation over the entire cell surface within 1–2 min (**Figures 7**, **1C, D**, **3C, D**). Due to their involvement in activation signaling at the local (but fast) mode, the signal transducers moesin, actin, and talin-1 might connect one by one to form a direct biophysical chain between the intracellular domains of PSGL-1 and β_2_ integrins (38, 40, 45, 48, 49).

This local activation mode provides evidence for the hypothesis that rolling neutrophils on E-selectin activate integrins to slow-down rolling on endothelial cells (8), but are not responsible for firm cell adhesion despite the presence of ICAM-1. In contrast, firm cell adhesion requires Ca^2+^-dependent global integrin activation *via* MAPK or non-MAPK pathways, in which cytosolic Ca^2+^ release or extracellular Ca^2+^ influx is involved (**Figures 3**, **4**).

Previous work on cell rolling on E-selectin in the presence of ICAM-1 has revealed a requirement for intact lipid rafts (8). Signaling *via* the MAPK pathway requires intact lipid rafts (24, 38), and so did the non-MAPK signaling in the fast (rather than the slow) mode because disruption of lipid rafts blocked P-selectin-induced cytosolic Ca^2+^ release and local integrin activation, but not integrin/ICAM-1 (GPIbα) axis-mediated extracellular Ca^2+^ influx (**Figures 6A, B**). The reason might arise from PSGL-1 partitioning lipid rafts (38), and PSGL-1 might coalesce small raft domains into a larger one to facilitate PSGL-1 signaling for neutrophils on immobilized P-selectin (50). The cytoskeleton has also been reported to be involved in PSGL-1 polarization, while the N-terminal ITAM of moesin serves as a bridge between Syk and a cytoplasmic PSGL-1 domain (24–26). We found that actin and moesin in the cytoskeleton were involved in the MAPK signaling cascade (25, 39) but also in non-MAPK signaling in the fast mode. Blockade of actin and moesin significantly inhibited Ca^2+^ responses and removed the effect of engagement with ICAM-1 or GPIbα on the tether lifetime for neutrophils on P-selectin (**Figure 6**). These results suggest that a bifurcation point of PSGL-1 signaling is located at a signal-transduction node: moesin–actin. In MAPK signaling cascades, Ca^2+^ bursts occurred before global activation of integrin over the entire cell surface (**Figure 2**) (34, 51), but both events had a similar timescale of a few minutes (**Figures 1**, **3**). This Ca^2+^ requirement did not exist in non-MAPK signaling in fast mode, so Ca^2+^ bursts were significant signals for integrin activation over the entire cell surface (52). We also showed regulation of the mechanical microenvironment on P-selectin-induced β_2_-integrin activation of neutrophils in flows. This statement is supported by force-triggered integrin activation (**Figure 1**) and force-enhanced cellular Ca^2+^ bursts in the presence and absence of ICAM-1 or GPIbα (**Figure 3**). This phenomenon might arise from the force-dependent interaction of PSGL-1 with P-selectin and integrin with its ligands (9, 12, 16, 31), force-induced cell membrane deformation, and skeletal rearrangement (53–55).

We herein just examined P-selectin-induced β_2_-integrin activation relevant to two specific neutrophil-adhesion events (tethering and firm adhesion) instead of rolling, and measured integrin activation over the entire cell surface in non-real-time, but observed cell tethering and Ca^2+^ bursts in real-time. This strategy led to underestimation of the loading-time requirement for integrin activation over the entire cell surface and lack of information on integrin activation for rolling cells. For example, in cell rolling, integrins close to ligated PSGL-1 would extend one-by-one. These freshly extended integrins would make cell roll slower by binding with their immobilized ligands (44) and would scatter gradually over the entire cell surface, while accumulation of signaling *via* the PSGL-1 axis as well as ICAM-1 or GPIbα engagement-induced integrin activation over the entire cell surface would occur. The present data showed that LFA-1 instead of Mac-1 mediated the firm adhesion and tether events of cells on P-selectin in the presence of ICAM-1 (**Supplementary Figures S1**, **S4**). Thus, we did not use anti-CD11b antibody in integrin activating and Ca^2+^ bursting of cells on P-selectin + ICAM-1, like the previous work for blood cell rolling (10). Besides, we used KIM127 instead of CBRM1/5 (another Mac-1 activation marker) in the present study. This was why we herein focused at whether β_2_ integrin was extended or not, in spite of that using CBRM1/5 to examine affinity state of Mac-1 over entire cell surface should be interested. And, the isolated neutrophils from whole blood were used in the present studies, because it was not feasible almost to observe the tether events of cells in whole blood in the flow chamber assay. The artifactual integrin activation from the cell isolation process might cause not only a down-estimation of mechanical stimulus time requirement for the local integrin activation but also an over-estimation of local integrin activation possibility. So, we will focus on the spatiotemporal characteristics of β_2_-integrin activation of neutrophils rolling on selectins *in vitro* (with whole blood) and *in vivo* in our future works.

## Conclusions

We demonstrated a model of P-selectin-induced β_2-_integrin activation for circulating human neutrophils. In this model, MAPK and non-MAPK signaling pathways participated in β_2_-integrin activation of neutrophils *via* the PSGL-1 axis. This involved a transition of signal transduction from a local (fast) mode to a global (slow) mode in the non-MAPK signaling cascade, and which might be a requirement for recruiting circulating leukocytes to inflammatory vascular sites. Our findings provide novel insights into β_2_-integrin activation but also cues in the development of molecular-targeted agents.

## Material and methods

### Antibodies and reagents

Two CD11a monoclonal antibodies (TS1/22 and IBL-6/2), goat anti-rat IgG (H+L) cross-adsorbed secondary antibody (Alexa Fluor 488), Hoechst 33342, and Fluo-4 acetoxymethyl (AM) ester were purchased from Invitrogen (Carlsbad, CA, USA). M1/70 and 2LPM19c (the CD11b monoclonal antibodies with and without Alexa Fluor 488) were obtained from eBioscience (San Diego, CA, USA) and Santa Cruz Biotechnology (Dallas, TX, USA), respectively. Purified mouse anti-human CD162 clone KPL-1 was sourced from BD Biosciences (Franklin Lakes, NJ, USA). Anti-CD11+CD18 antibody (mAb24) was from Abcam (Cambridge, UK). An Alexa Fluor 594-labeled IgG kit was purchased from Invitrogen. The KIM127 monoclonal antibody to the human β_2_-subunit was purified from a hybridoma supernatant (American Type Culture Collection, Manassas, VA, USA). Bovine serum albumin (BSA), Histopaque 1077, Histopaque 1119, Ficoll PM 400, DMSO, LaCl_3_, 2-APB, MβCD, piceatannol, staurosporine, and MNS were from MilliporeSigma (Burlington, MA, USA). An actin inhibitor, cytochalasin B, was sourced from Aladdin (Shanghai, China). ACK lysing buffer and 4-(2-hydroxyethyl)-1-piperazineethanesulfonic acid (HEPES) were obtained from Gibco (Grand Island, NY, USA). Recombinant human P-selectin/Fc chimera, recombinant human ICAM-1/Fc chimera, and recombinant human CD42b (GPIbα) were from R&D Systems (Minneapolis, MN, USA). Hank’s balanced salt solution (HBSS) and phosphate-buffered saline (PBS) were obtained from Gibco. NaCl, KCl, CaCl_2_, and MgCl_2_ were from purchased DM Reagents (Tianjin, China). Glucose was sourced from GBCBIO Technologies (Guangzhou, China). All reagents were of the highest purity available.

### Neutrophil isolation

The study protocol was approved by the Research Ethics Committee of Guangzhou First People’s Hospital within South China University of Technology (Guangzhou, China) and aligned with the Declaration of Helsinki 1964 and its later amendments. Written informed consent was provided from all healthy volunteers.

Sodium-citrated whole human blood was obtained from healthy volunteers. Human neutrophils were isolated by a density-gradient separation method, as described previously (28). Briefly, Histopaque 1119 solution (3 mL) and Histopaque 1077 solution (3 mL) were layered gently onto the bottom of a 15-mL conical centrifugation tube. Then, 6 mL of fresh blood was added slowly to the top of the tube. The mixed solution was centrifuged at 700 × *g* for 30 min at room temperature. Neutrophils were collected from the appropriate density layer (granulocyte layer) and washed with HBSS (10 mL) without Ca^2+^ or Mg^2+^. After washing, the tube was centrifuged at 400 × *g* for 10 min at room temperature. The supernatant was removed, and erythrocyte-lysing buffer (ACK lysis buffer; 3 mL) was used for resuspension. Subsequently, the tube was shaken gently for 10 min at 37°C. Then, neutrophils were washed with HBSS (10 mL). After centrifugation at 400 × *g* for 10 min at room temperature, the supernatant was discarded, and the neutrophil pellet was resuspended in HBSS at room temperature to test cell viability based on the exclusion of trypan-blue dye. The obtained preparations were >95% pure and viable.

### Flow-chamber assay

Five protein solutions were created by dissolving their respective dry powders into PBS (40 μL): P-selectin/Fc (10 μg/mL); P-selectin/Fc (10 μg/mL) plus ICAM-1/Fc (5 μg/mL); P-selectin/Fc (10 μg/mL) plus GPIbα (40 μg/mL); ICAM-1 (5 μg/mL); GPIbα (40 μg/mL). Another five protein solutions were mixed by dissolving P-selectin (30 ng/mL) alone or combining it with ICAM-1 (500 ng), GPIbα (20 μg/mL), KIM127 (1 μg/mL), or mAb24 (1 μg/mL) into PBS (40 μL), respectively. Each of these solutions was used to functionalize the parallel plate flow chamber (PPFC) (length × width × height = 20 × 5 × 0.254 mm^3^, see **Supplementary Figure S8**), as described previously (31). The solution (40 μL) was added to a coating region (5 mm × 5 mm), which was marked in the cover-slide center and held by a hollowed silicon gasket on the bottom surface of a petri dish (Corning Glass Works, Corning, NY, USA), and incubated overnight at 4°C. The protein-coated region was washed thrice with HBSS containing 2 % BSA (*w/v*) to remove excess unabsorbed proteins and then incubated within the same solution for 1 h at room temperature to block nonspecific adhesion of cells. Selected densities for P-selectin, ICAM-1, and GPIbα of 10, 5, and 40 μg/mL supported firm adhesion instead of a transient tether stop. Of all five functionalized substrates of PPFC, each was used to test the firm adhesion and Ca^2+^ response of cells under fluid shear stress (0.2, 0.6, or 2.0 dyne/cm^2^). Three substrates coated with P-selectin alone or with either ICAM-1 or GPIbα were used to test integrin activation over the entire surface of firmly adhered cells in flow.

Isolated neutrophils were resuspended in HBSS containing 2% BSA (*w/v*) and Ca^2+^ (1.5 mmol/L) at a final cell concentration of 1.0×10^6^ cells/mL. To test whether cell adhesion was specific for P-selectin, ICAM-1, and GPIbα, each of the five substrates mentioned above was also used in the PPFC experiment. Neutrophils were pretreated with MgCl_2_ (10 mmol/L), mouse anti-human CD162 clone KPL-1 (20 μg/mL), monoclonal anti-LFA-1 monoclonal antibody (TS1/22, 10 μg/mL), and anti-Mac-1 monoclonal antibodies (2LPM19c, 10 μg/mL). In each specific experiment, a neutrophil suspension (5×10^5^ cells/mL) was perfused over each substrate for 1 min at 0.2 dyne/cm^2^. Images were recorded at 100 fps by a digital complementary metal oxide semiconductor (CMOS) camera (ORCA-Flash4.0 V3; Hamamatsu, Hamamatsu, Japan) coupled to an inverted microscope. A “firm cell-adhering event” was defined as a cell movement of distance <10 μm in 1 min. All adhesion events in the view window over a 7-min observation were counted. The number of firmly adhered cells and the total number of all cells observed near the flow-chamber floor were counted by Image-Pro Plus 6.0 (US National Institutes of Health, Bethesda, MD, USA) and Excel™ 2010 (Microsoft, Redwood, WA, USA). The ratio of firmly adherent cells was calculated by dividing the number of firmly adherent cells by the total number of all cells on the flow-chamber floor.

We wished to examine integrin activation over the entire surface of cells on immobilized P-selectin with or without ICAM-1 or GPIbα in flow. The cell suspension was perfused over PPFC substrates with a syringe pump (PHD22/2000; Harvard Apparatus, Holliston, MA, USA) under a wall shear stress 0.2 dyne/cm^2^ for 0.5, 1.0, 2.0, or 3.0 min from emergence of the first firmly adherent cell. A cell suspension (40 μL) was dropped gently onto the functionalized PPFC bottom for 0.5 or 3.0 min for the static condition. In an inhibition experiment, neutrophils were preincubated with a chemical inhibitor of inositol 1,4,5-trisphosphate (IP_3_) receptors, 2-APB (100 μmol/L), for 8 min or an inhibitor of Ca^2+^ channels on cell membranes, LaCl_3_ (10 μmol/L), for 30 min. This was done to detect the effect of Ca^2+^ signals on integrin activation. The firmly adherent cells on the PPFC bottom were washed with HBSS containing 2 % BSA (*w/v*) to remove free cells. Next, they were fixed in 4 % paraformaldehyde for 30 min at room temperature, followed by blockade with HBSS containing 2 % BSA overnight at 4°C. To identify extended integrins, fixed cells on substrates coated with P-selectin alone or with ICAM-1 were incubated in turn with the monoclonal antibody CD11a (IBL-6/2) (5 µg/mL), goat anti-rat secondary antibody Alexa Fluor 488 (10 µg/mL), and Alexa Fluor 594–labeled KIM127 monoclonal antibody (20 µg/mL) at room temperature for 1 h. Meanwhile, fixed cells on substrates coated with P-selectin alone or with GPIbα were incubated in turn with Alexa Fluor 488-labeled monoclonal antibody CD11b (M1/70) (10 µg/mL) and Alexa Fluor 594-labeled KIM127 monoclonal antibody (20 µg/mL) for 1 h at room temperature. Nuclei were stained with Hoechst 33342 (12.5 µmol/L) for 30 min at room temperature. Images were acquired under the objective lens (60 × 0.70 NA) on an inverted microscope (Eclipse Ti2; Nikon, Tokyo, Japan) using the digital CMOS camera. To quantify assays, ten random fields of each group were analyzed by ImageJ, and the relative KIM127-relative cellular fluorescence intensity was evaluated by (FI_C_-FI_B_)/FI_B_ = FI_C_/FI_B_ -1, where FI_C_ and FI_B_ were the KIM127-related cellular and background fluorescence density per unit surface, respectively (**Figures 1C, D**). Next, confocal laser scanning microscopy was done on the focal plane near the cell midsection using an SP8 system (Leica, Wetzlar, Germany) to uncover the pattern of extended integrin contribution on cell (**Figures 1A, B**).

A cell had β_2_-integrin extension if its KIM127-related fluorescence intensity was high in comparison with its background. Two parameters were used to quantify the temporal characteristics of β_2_-integrin extension *via* different durations of mechanical stimuli: (i) the relative density of extended-β_2_ integrins (KIM127-related fluorescence intensity of a cell over that of the background minus one); (ii) cell activation ratio (proportion of β_2_ integrin extension-positive cells in all firmly adherent cells), which was normalized by dividing it by its value in the absence of a mechanical load.

### Measurement of the Ca^2+^ response

Measurement of the Ca^2+^ response was done as described previously (28). Briefly, isolated neutrophils were resuspended in loading buffer (1 % BSA (*w/v*), HEPES (20 mmol/L), and glucose (20 mmol/L) in PBS (pH 7.4) at 1.0×10^6^ cells/mL. The monochrome Ca^2+^-sensitive dye Fluo-4 AM was applied to detect intracellular Ca^2+^ at longer times. For real-time monitoring of changes in intracellular Ca^2+^ levels, Fluo-4 AM (1 μmol/L) was added to a neutrophil suspension for 30 min at 37°C. After centrifugation at 400 × *g* for 10 min at 37°C, the neutrophils were resuspended in loading buffer without Fluo-4 AM for an additional 30 min at 37°C until use. To block IP_3_ receptors and Ca^2+^ channels in cell membranes, neutrophils were incubated with 2-APB (100 μmol/L, 8 min) or LaCl_3_ (10 μmol/L, 30 min), respectively. Also, neutrophils were incubated with a disruptor of lipid rafts (MβCD, 5 mmol/L, 5 min), actin disruptor (cytochalasin B, 5 μg/mL, 5 min), Syk inhibitor (piceatannol, 20 μmol/L, 30 min), moesin inhibitor (staurosporine, 0.01 μmol/L, 30 min), talin inhibitor (MNS (56, 57), 20 μmol/L, 3 min), and an equal volume of DMSO (vehicle control), respectively. These diversely treated neutrophils were used to detect the actors in Ca^2+^ signaling but also in the tether events of cells on immobilized P-selectin.

Each of the five substrates mentioned above was used in the PPFC experiment for examining the Ca^2+^ response of cells on immobilized P-selectin, ICAM-1, or GPIbα. These pretreated cells were resuspended at 1×10^6^ cells/mL in imaging buffer (NaCl (110 mmol/L), KCl (10 mmol/L), glucose (10 mmol/L), HEPES (30 mmol/L), CaCl_2_ (1.5 mmol/L), 1 % BSA (*w/v*), 12 % Ficoll (*w/v*), pH 7.4). A neutrophil suspension was perfused over no agent, 1 % BSA, or each of the above-mentioned PPFC substrates under different wall shear stress (0.2, 0.6, or 2.0 dyne/cm^2^) for 7 min. Fluorescence images of firmly adherent cells were acquired at 20 fps by the CMOS camera coupled with the inverted microscope running NIS-Elements AR 5.01.00 for 64-bit software (Nikon). The fluorescence intensity of a cell was normalized using the equation F_IN_ = (F_IC_ – F_IB_)/F_IB_, where F_IB_ is the mean background fluorescence intensity (mean value of four fluorescence-intensity values from four equidistant round domains of 36π μm^2^ around the cell at a distance of 24 μm), and F_IN_ and F_IC_ are the normalized and mean cell fluorescence intensities, respectively. The Ca^2+^ burst ratio and delay time of Ca^2+^ bursts were used to characterize Ca^2+^ signaling in neutrophils. The Ca^2+^ burst ratio was the percentage of Ca^2+^ burst events in all firmly adherent cells in the viewing field over an observation period of 7 min. The delay time of Ca^2+^ bursts was the duration from emerging to Ca^2+^ bursts of firmly adherent cells on the substrate.

### Measurement and analyses of the tether lifetime

The observation and analyses of a tether event were done as described previously (31). Briefly, the PPFC bottom was functionalized by coating with P-selectin (30 ng/mL in 40 μL of PBS) alone or with ICAM-1 (500 ng/mL), GPIbα (20 μg/mL), KIM127 (1 μg/mL), and mAb24 (1 μg/mL), respectively, as mentioned above. P-selectin density of 30 ng/mL was selected to support a transient stop of tethering instead of firm adhesion or stable rolling. All five newly functionalized PPFC substrates were employed to test instantaneous integrin activation of neutrophils on immobilized P-selectin. Isolated neutrophils were suspended in HBSS containing 2 % BSA (*w/v*) and Ca^2+^ (1.5 mmol/L) at a final cell concentration of 1.0×10^6^ cells/mL. The cell suspension was perfused over each of the five newly functionalized substrates under various values of wall shear stress (0.1–0.45 dyne/cm^2^) for 5 min. A tether event (stop between two consecutive movements of flowing cells) and the stop time of cells on substrates were recorded at 100 fps by the digital CMOS camera coupled to an inverted microscope (Axio Observer A1; Zeiss, Oberkochen, Germany) and analyzed using Image-Pro Plus 6.0 and Excel™ 2010. The lifetime of ≥100 tethering events was analyzed and averaged for each experiment. Meanwhile, as described above, neutrophils, which had been pretreated with MβCD, cytochalasin B, piceatannol, staurosporine, and MNS as well as DMSO (vehicle control), respectively, were also used to detect the actor in tether events. To examine if a tether event was specific for P-selectin, LFA-1, and Mac-1, neutrophils were pretreated by KPL-1 (20 μg/mL), TS1/22 (10 μg/mL), or 2LPM19c (10 μg/mL). The tethering adhesion ratio was calculated by dividing the number of tethered cells by the total number of all cells observed near the flow-chamber floor for 1 min, and the tether force was estimated using 125 pN/(dyne/cm^2^), a conversion from wall shear stress to tether force (58).

### Statistical analyses

Statistical significance was analyzed by two-way (or one-way) analysis of variance (ANOVA) for multiple comparisons with the *post-hoc* Bonferroni test in SPSS 24 (IBM, Armonk, NY, USA). All error bars represent the mean plus or minus standard error of the mean based on several independent experiments indicated in the figure legends.

## Data availability statement

The original contributions presented in the study are included in the article/**Supplementary Material**. Further inquiries can be directed to the corresponding authors.

## Ethics statement

The studies involving human participants were reviewed and approved by The Research Ethics Committee, Guangdong General Hospital, Guangzhou, China. The patients/participants provided their written informed consent to participate in this study. Written informed consent was obtained from the individual(s) for the publication of any potentially identifiable images or data included in this article.

## Author contributions

JW, YF, XS, BH conceived the experiments, which were performed by XS, BH, YP, HW and YJ. XS performed flow chamber assay. XS and BH performed calcium response measurement, with the helping of HW and YJ. XS and YP performed tether lifetime measurement and analysis. YF, XS, BH, YP, HW and YJ analyzed the data. JW, XS, BH, YP, JF, YJ, PG, JL, HW, YL, and QL wrote the manuscript. JW, YF and BH secured the funding. All authors contributed to the article and approved the submitted version.

## Funding

This work was supported by the National Natural Science Foundation of China [Grant Nos. 11432006 (JW), 12072117 (JW), 12172137 (YF), 82170566 (BH), and 82000518 (BH)].

## Conflict of interest

The authors declare that the research was conducted in the absence of any commercial or financial relationships that could be construed as a potential conflict of interest.

## Publisher’s note

All claims expressed in this article are solely those of the authors and do not necessarily represent those of their affiliated organizations, or those of the publisher, the editors and the reviewers. Any product that may be evaluated in this article, or claim that may be made by its manufacturer, is not guaranteed or endorsed by the publisher.
