# Supplementary material for: Spatiotemporal characteristics of P-selectin-induced β2 integrin activation of human neutrophils under flow
Source: Front Immunol. 2022 Nov 10;13:1023865. doi: 10.3389/fimmu.2022.1023865 (PMC9692129; doi:10.3389/fimmu.2022.1023865)
Supplement: Supplementary file 1 [file DataSheet_1.pdf]

Supplementary Material

Supplementary Figures

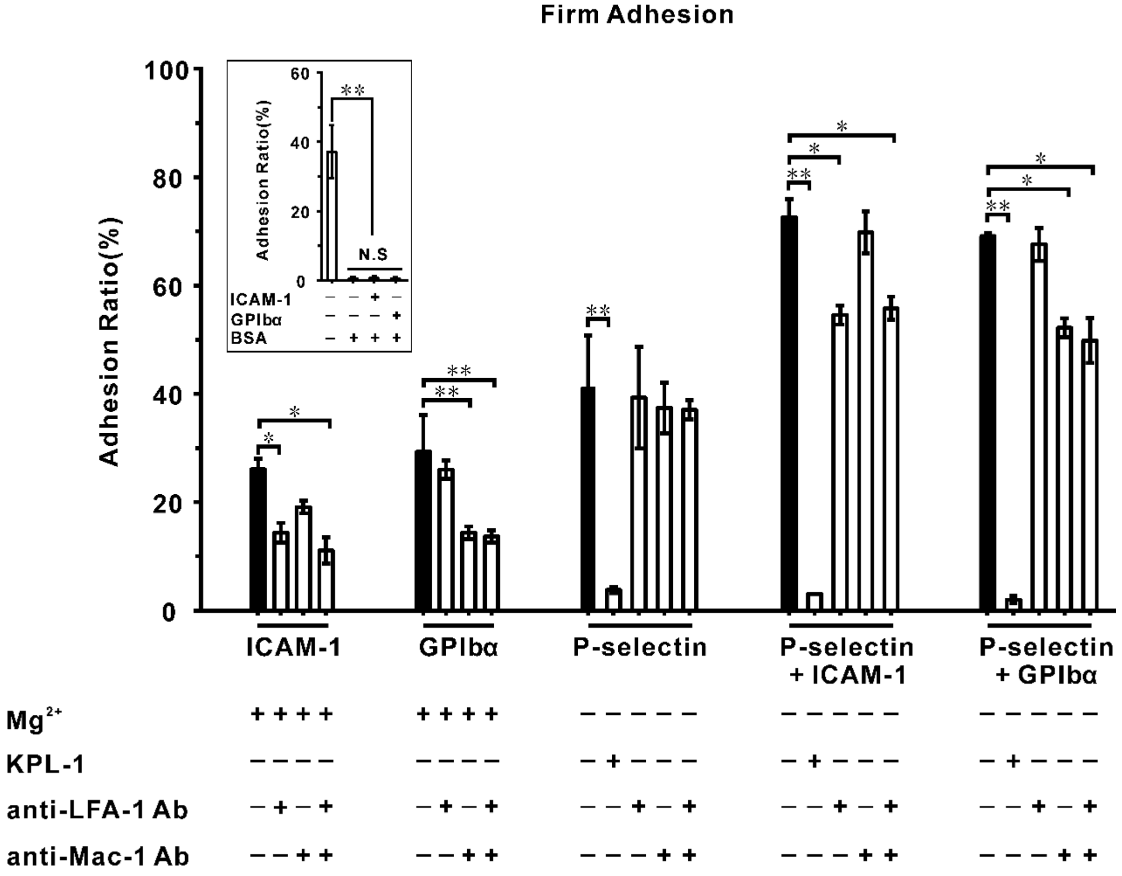

Supplementary Figure 1. Specific firm adhesion of neutrophils on P-selectin. Human neutrophils were treated with BSA, Mg^{2+}, Anti-PSGL-1 blocking Ab (KPL-1), Anti-LFA-1 blocking Ab (TS1/22), and Anti-Mac-1 blocking Ab (2LPM19c) before they were poured into the functionalized flow chamber. Data were the firm adhesion ratios of neutrophils on immobilized P-selectin combined with ICAM-1, GPIbα, and nothing under the shear stress of 0.2 dyne/cm^2, representing the mean ± SEM in three different independent experiments and analyzed by two-way ANOVA for multiple comparisons. The inset exhibited the data of the firm adhesion ratios for neutrophils on immobilized ICAM-1, GPIbα, and nothing with or without treatment of BSA under the shear stress of 0.2 dyne/cm^2. The significant level of difference was shown by P-value, * $P < 0.05$, ** $P < 0.001$, N.S. for no significant difference.

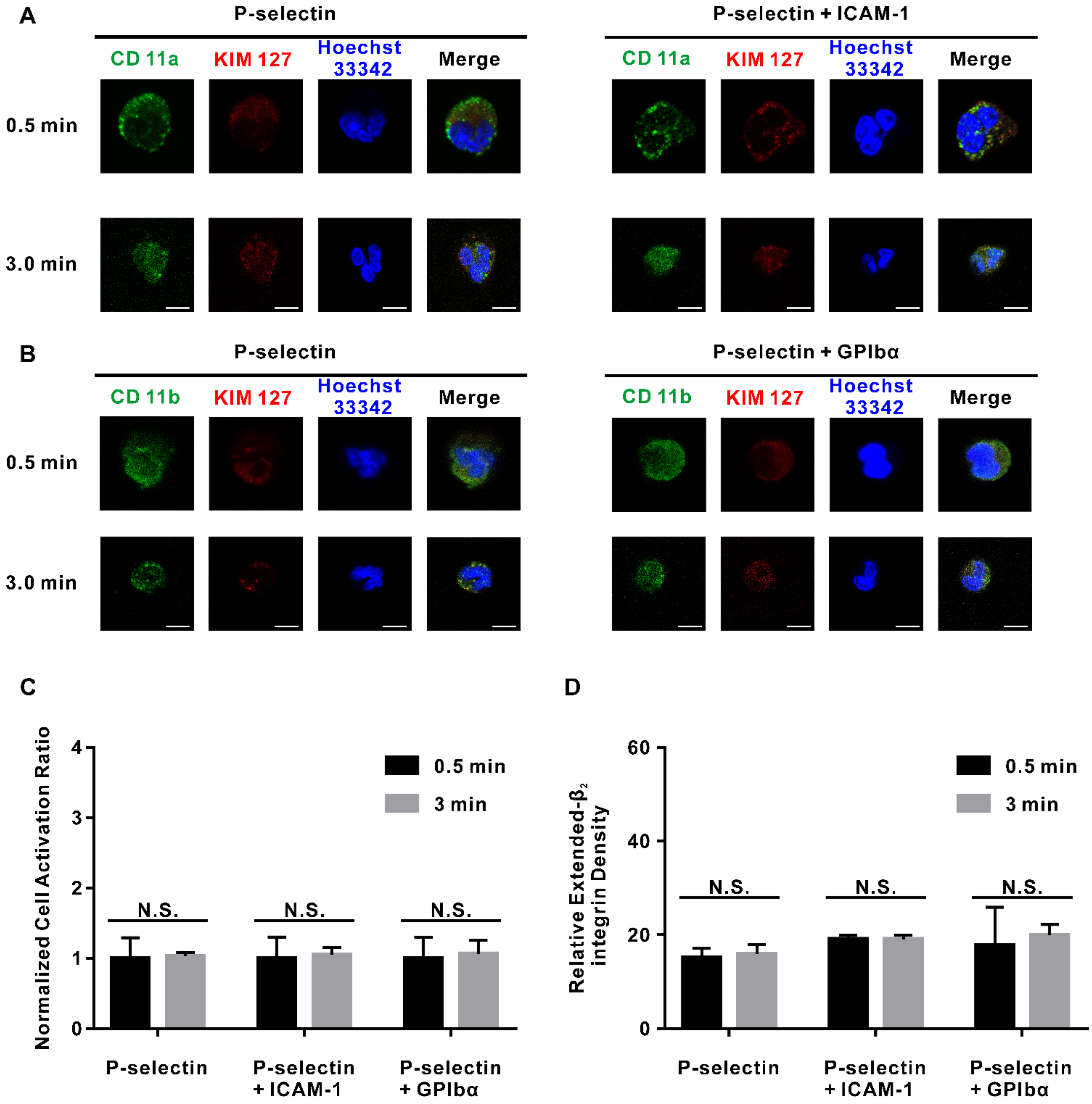

Supplementary Figure 2. Activation ratio and extended β2 integrin intensity of human neutrophils under static state. Human neutrophils were held on substrates coated with P-selectin with or without ICAM-1, GPIbα for 0.5 min and 3 min on the static condition, then fixed and stained with anti-CD11a Ab, anti-CD11b Ab, β_2 subunit extended indicator KIM127 Ab, Hoechst 33342 for nuclei. (**A, B**) Representative patterns and their merged images of Hoechst 33342-stained nuclei (blue) and β_2 integrin bound with anti-CD11a Ab (green), anti-CD11b Ab (green), and KIM127(red), using confocal laser scanning microscopy was done using an SP8 system (Leica, Wetzlar, Germany). The scale bars = 5 µm for each image. The normalized cell activation ratio (C) and relative extended-

β_2 integrin density (D) were acquired from images captured by an inverted microscope (ECLIPSE Ti2; Nikon), using an ORCA-Flash4.0 V3 digital CMOS camera. The cell activation ratio on static condition for 0.5 min was represented for the normalized activated ratio =1. The data represented the mean ± SEM from three independent experiments. Ten random images of each experiment were analyzed by ImageJ. Statistical significance was analyzed by two-way ANOVA for multiple comparisons. The significant level of difference is shown by *P*-value, N.S. for no significant difference.

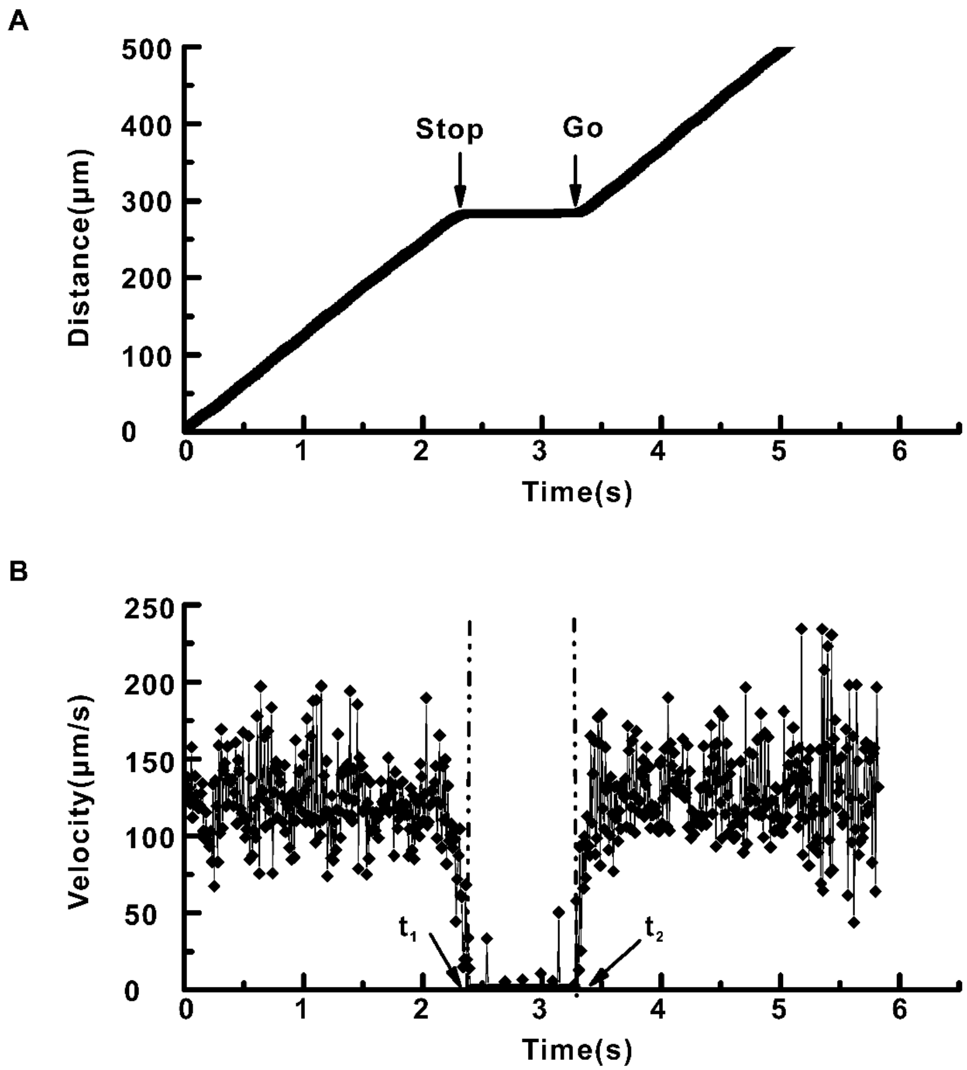

Supplementary Figure 3. A typical time course of movement of flowing neutrophils on the flow chamber bottom under wall shear stress of 0.2 dyne/cm^2. Tether event (stop between two consecutive movements of the flowing cell) and stop time (or tether lifetime) of the cell on the substrate could be read from this time-course of (A) accumulative distance of cell movement and (B) instantaneous velocity of the flowing cell. Tether lifetime was the interval timing between go time (t_2) and stop time(t_1).

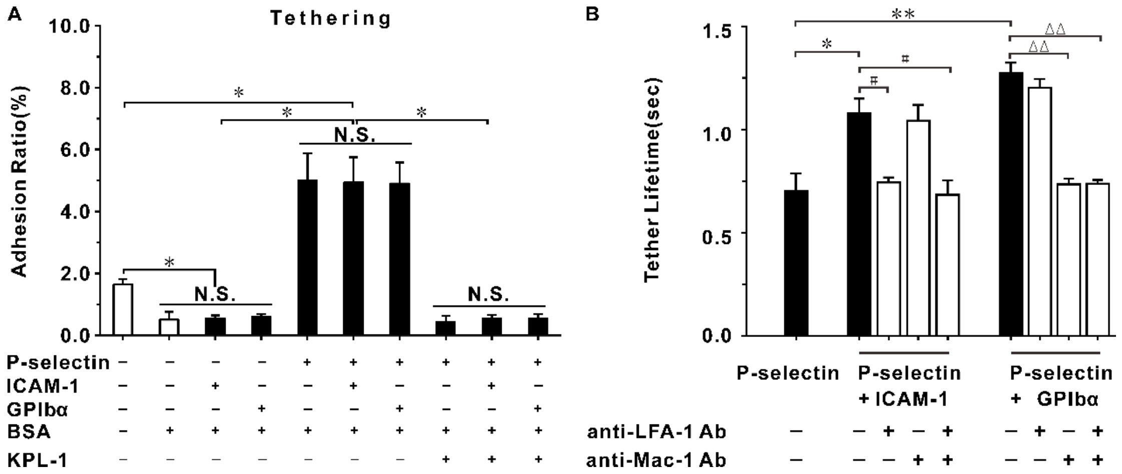

Supplementary Figure 4. Tethering of neutrophils to P-selectin instead of ICAM-1 or GPIbα is specific. And extended integrin on cell contact area was responsible for ICAM-1 or GPIbα engagement-mediated increase of tether lifetime. (A) Human neutrophils, which were pretreated by BSA (for control) and anti-PSGL-1 blocking Ab (KPL-1), were poured over the PPFC substrates coated with P-selectin, ICAM-1, GPIbα, and nothing under the shear stress of 0.2 dyne/cm^2. The tethering ratio of neutrophils represented the mean ± SEM in three different independent experiments and was analyzed by two-way ANOVA for multiple comparisons. The significant level of difference was shown by P-value, * $P < 0.05$, ** $P < 0.001$, N.S. for no significant difference. (B) Human neutrophils were pretreated by BSA, anti-LFA-1 blocking Ab, and anti-Mac-1 blocking Ab before pouring over substrates coated with P-selectin with or without ICAM-1 and GPIbα at wall shear stress of 0.2 dyne/cm^2. The data of lifetime represented the mean ± SEM in three independent experiments and analyzed by two-way ANOVA for multiple comparisons. The significant level of difference from the P-selectin group was shown by P-value, * $P < 0.05$, ** $P < 0.001$. The significant level of difference from the control group on the same substrates was shown by P-value, # Δ for $P < 0.05$, ## ΔΔ for $P < 0.001$.

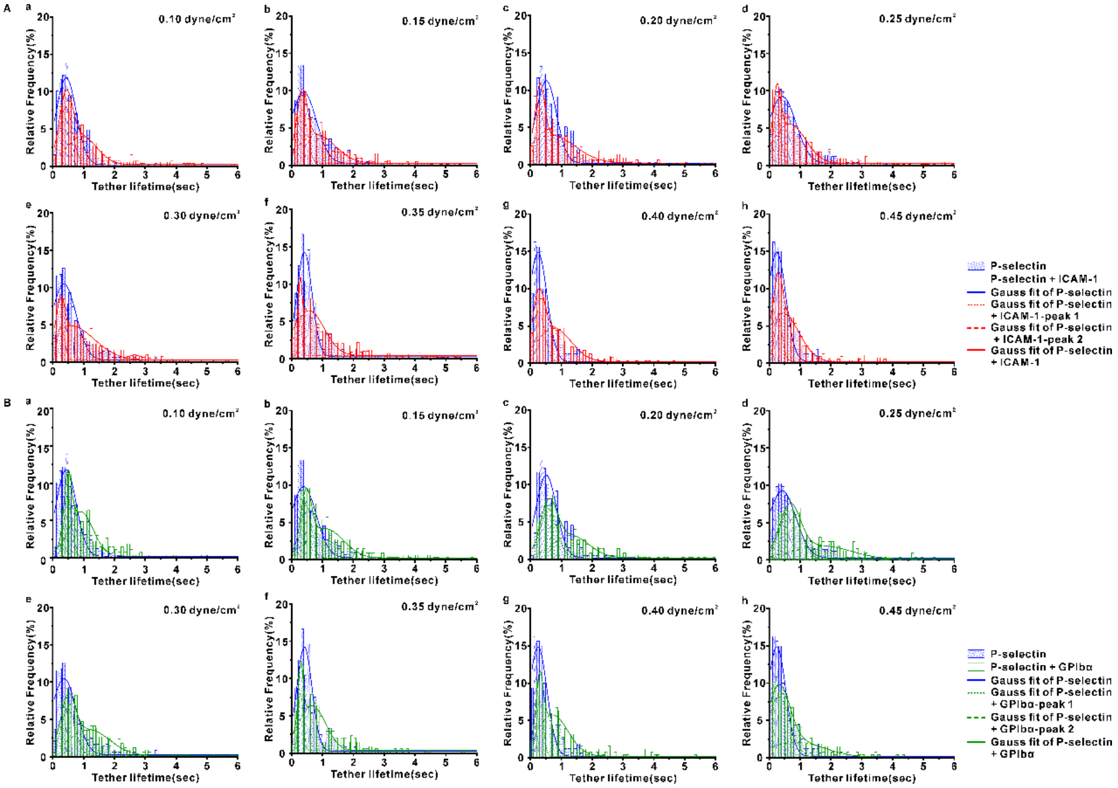

Supplementary Figure 5. Patterns of tether lifetime distribution of human neutrophils on P-selectin alone or combined with ICAM-1 or GPIbα under various wall shear stresses. Human neutrophils were poured over substrates coated with P-selectin alone (blue) or combined with ICAM-1 (red) (A) or plus GPIbα (green) (B) under various wall shear stresses from 0.1 to 0.45 dyne/cm^2. Tether lifetime distributions of neutrophils on P-selectin combined with ICAM-1 (A)or GPIbα (B) were fitted by Gauss distribution (blue solid line for P-selectin, red line for P-selectin+ICAM-1, or green line for P-selectin+GPIbα). The patterns of tether lifetime distribution for human neutrophils on P-selectin combined with ICAM-1 (A) or GPIbα (B) had two peaks respectively: one (red dot line) was regarded as the contribution from P-selection alone, and one (red dash line) from ICAM-1 engagement in panel A; one (green dot line) were regarded as the contribution from P-selection alone, and one (green dash line) from GPIbα engagement in panel B. The data collected at least 250 events for each set in at least three independent experiments.

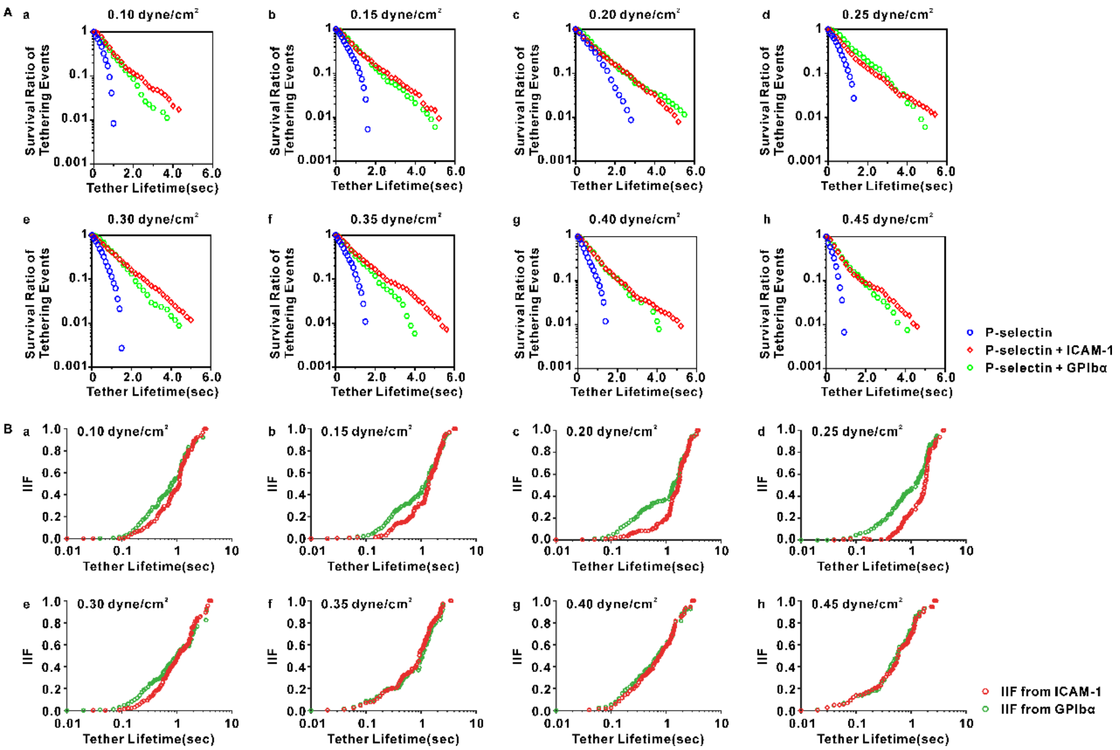

Supplementary Figure 6. Plots of survival ratio of tethering events against tether lifetime and variation of integrin-involved fraction (IIF) versus tether lifetime for cells on different substrates at wall shear stress of 0.20 dyne/cm^2. (A) Variation of survival ratios of tethering events versus tether lifetime for cells on P-selectin in the existence of ICAM-1 (red), GPIbα (green) or nothing (blue) at wall shear stresses of 0.1(**a**), 0.15(**b**), 0.2(**c**), 0.25(**d**), 0.3(**e**), 0.35(**f**), 0.4(**g**) and 0.45(**h**) dyne/cm^2. ICAM-1 or GPIbα engagement led to significant increments of survival ratio of tether events for each wall shear stress. The difference between survival ratios from ICAM-1 and GPIbα engagement was very small, in comparison with the ICAM-1 and GPIbα engagement-induced survival ratio increments. (B) Plots of integrin-involved fraction (IIF) against tether lifetime for cells on P-selectin in the existence of ICAM-1 (red) or GPIbα (green) at wall shear stresses of 0.1(**a**), 0.15(**b**), 0.2(**c**), 0.25(**d**), 0.3(**e**), 0.35(**f**), 0.4(**g**) and 0.45(**h**) dyne/cm^2. IIF increased with tether lifetime behind the latency (IIF = 0.1) of about 0.1 seconds and IIF reached one at about 3 seconds for each wall shear stress.

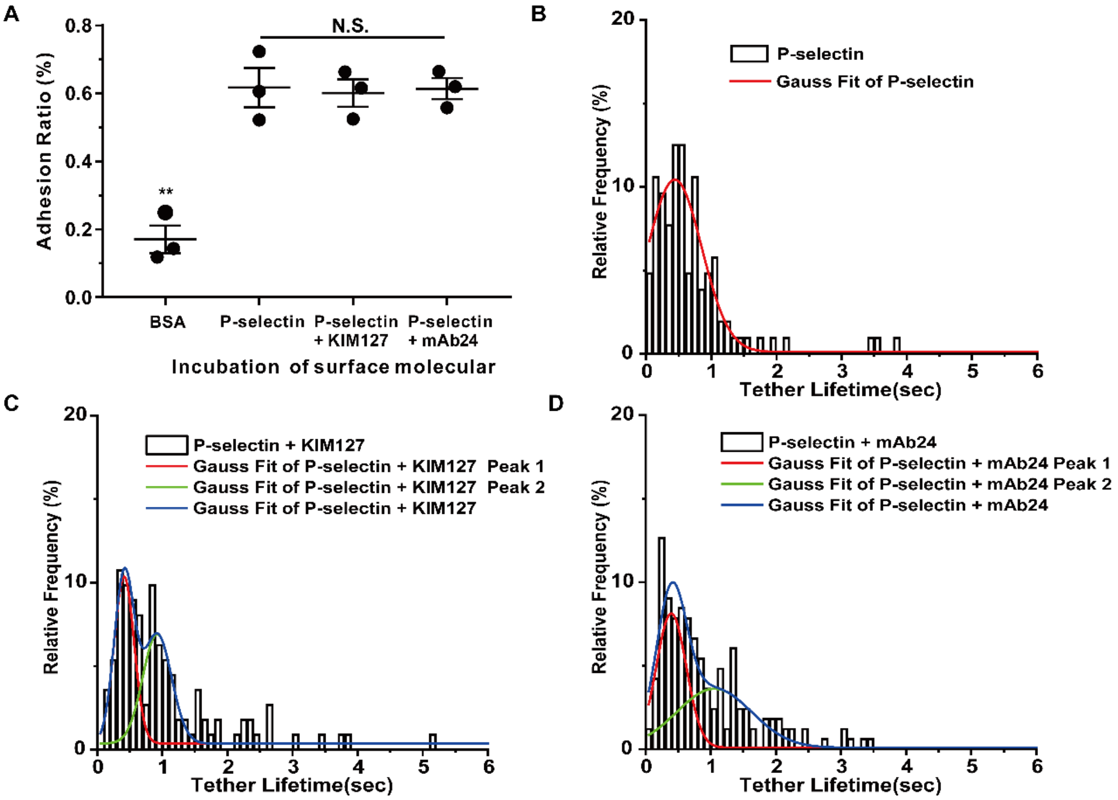

Supplementary Figure 7. P-selectin-induced an extending but head-closed instead conformation of β2 integrin (A) Adhesion ratio of human neutrophils tethering to the indicated substrates at 0.2 dyne/cm^2. The substrates were coated with 2 % BSA, P-selectin, and P-selectin mixed with β2 integrin extension sensitive recognition antibody KIM127 (recognized the β subunit I-EGF2 domain) or mAb24 (recognized the β subunit I-like domain) to be functionalized. Data were presented as the mean ± SEM from at least three independent experiments and analyzed by one-way ANOVA for multiple comparisons. The significant level of difference is shown by *P*-value, N.S. represented for no significant difference, * $P < 0.05$, ** $P < 0.001$. (B-D) The frequency distribution of human neutrophils' tether lifetime was analyzed at 0.2 dyne/cm^2. Human neutrophils were poured into the functionalized flow chamber coated with P-selectin (white column, panel B), P-selectin, and KIM127 (white column, panel C), or P-selectin and mAb24 (white column, panel D). The frequency distribution of tether lifetime was fitted by Gauss of P-selectin (red solid line, panel B), P-selectin and KIM127 (blue solid line in panel C), or P-selectin and mAb24 (blue solid line in panel D). The frequency distribution of human neutrophil's tether lifetime on the plate coated with P-selectin mixed with KIM127 (panel C), or P-selectin mixed with mAb24 (panel D) were fitted into two peaks, which were peak 1 (red solid line) and peak 2 (green solid line). Data were presented from at least three independent experiments. The tether lifetime of at least 104 tethering events for each set was analyzed.

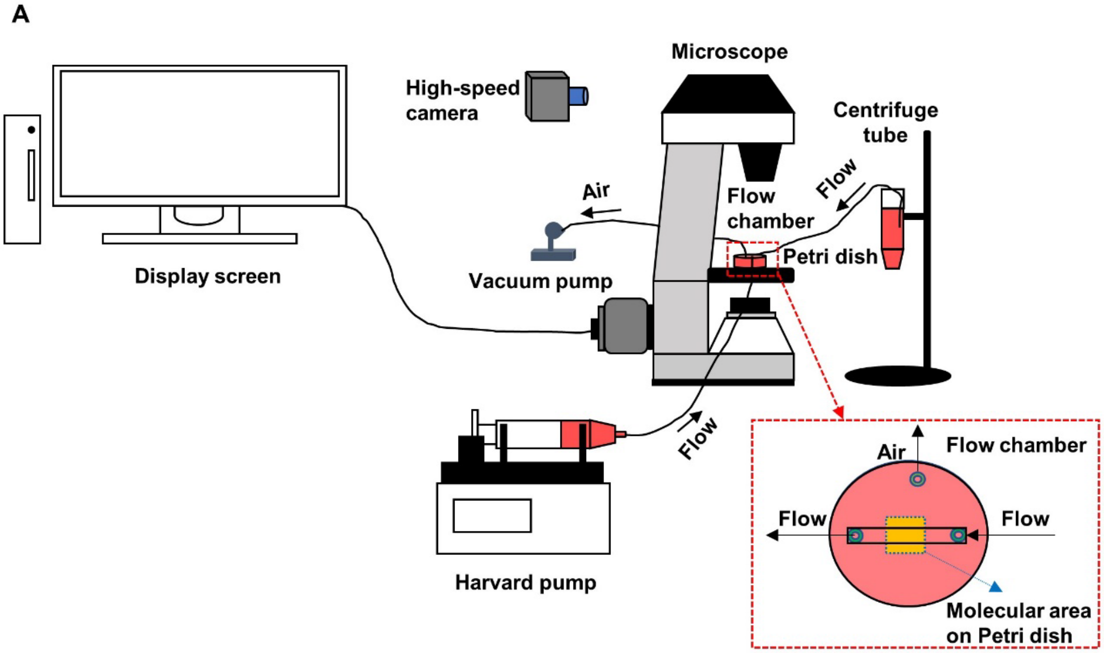

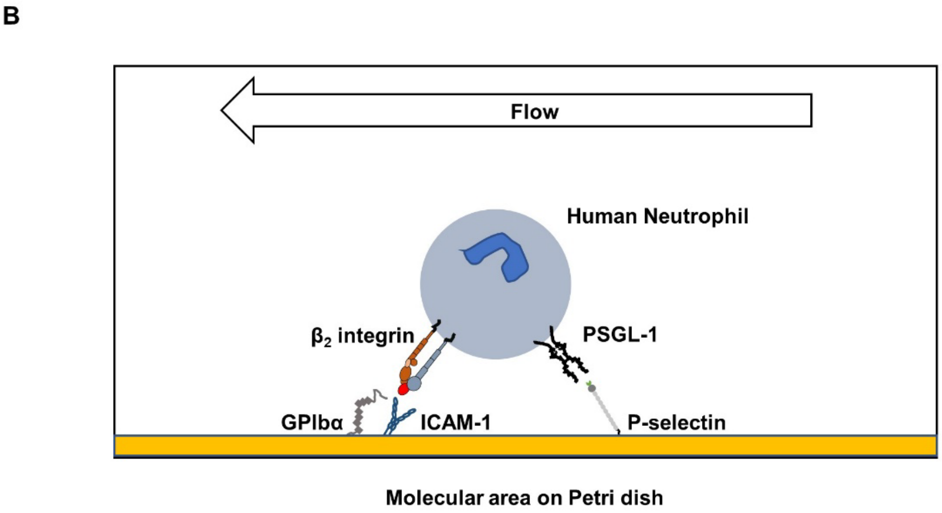

Supplementary Figure 8. The schematic of the parallel-plat flow chamber (PPFC) setup. (A) The schematic of the parallel-plat flow chamber (PPFC) setup. (B) The profile of molecular area on Petri dish section with flow chamber.
